# Silica Coated Iron/Iron Oxide Nanoparticles as a Nano-Platform for T_2_ Weighted Magnetic Resonance Imaging

**DOI:** 10.3390/molecules24244629

**Published:** 2019-12-17

**Authors:** Paul Mathieu, Yannick Coppel, Marc Respaud, Quyen T. Nguyen, Sébastien Boutry, Sophie Laurent, Dimitri Stanicki, Céline Henoumont, Fernando Novio, Julia Lorenzo, David Montpeyó, Catherine Amiens

**Affiliations:** 1 CNRS, LCC (Laboratoire de Chimie de Coordination), 205 route de Narbonne, BP 44099, CEDEX 4, F-31077 Toulouse, France; paul.mathieu@lcc-toulouse.fr (P.M.); yannick.coppel@lcc-toulouse.fr (Y.C.); thiquyen.nguyen@lcc-toulouse.fr (Q.T.N.); 2 Université de Toulouse, UPS, INPT, CEDEX 4, F-31077 Toulouse, France; 3 LPCNO, INSA, 135 Avenue de Rangueil, CEDEX 4, 31077 Toulouse, France; 4 Department of General, Organic and Biomedical Chemistry, NMR and Molecular Imaging Laboratory, University of Mons, 19 Avenue Maistriau, B-7000 Mons, Belgium; Sebastien.BOUTRY@umons.ac.be (S.B.); sophie.laurent@umons.ac.be (S.L.); dimitri.stanicki@umons.ac.be (D.S.); Celine.HENOUMONT@umons.ac.be (C.H.); 5 Center for Microscopy and Molecular Imaging (CMMI), Université de Mons (UMONS), B-6041 Charleroi, Belgium; 6 Departament de Química, Universitat Autònoma de Barcelona (UAB), Campus UAB, 08193 Cerdanyola del Vallès, Barcelona, Spain; fernando.novio@icn2.cat; 7 Institut de Biotecnologia i Biomedicina, Departament de Bioquimica i de Biologia Molecular, Universitat Autonoma de Barcelona, 08193 Bellaterra, Spaindavid.montpeyo@uab.cat (D.M.)

**Keywords:** nanomaterials, nanochemistry, surface functionalization, MRI, toxicity

## Abstract

The growing concern over the toxicity of Gd-based contrast agents used in magnetic resonance imaging (MRI) motivates the search for less toxic and more effective alternatives. Among these alternatives, iron–iron oxide (Fe@FeOx) core-shell architectures have been long recognized as promising MRI contrast agents while limited information on their engineering is available. Here we report the synthesis of 10 nm large Fe@FeOx nanoparticles, their coating with a 11 nm thick layer of dense silica and functionalization by 5 kDa PEG chains to improve their biocompatibility. The nanomaterials obtained have been characterized by a set of complementary techniques such as infra-red and nuclear magnetic resonance spectroscopies, transmission electron microscopy, dynamic light scattering and zetametry, and magnetometry. They display hydrodynamic diameters in the 100 nm range, zetapotential values around −30 mV, and magnetization values higher than the reference contrast agent RESOVIST^®^. They display no cytotoxicity against 1BR3G and HCT116 cell lines and no hemolytic activity against human red blood cells. Their nuclear magnetic relaxation dispersion (NMRD) profiles are typical for nanomaterials of this size and magnetization. They display high r_2_ relaxivity values and low r_1_ leading to enhanced r_2_/r_1_ ratios in comparison with RESOVIST^®^. All these data make them promising contrast agents to detect early stage tumors.

## 1. Introduction

Contrast agents in magnetic resonance imaging (MRI) may be divided into two categories [1]. So-called T_1_ contrast agents decrease the longitudinal relaxation rate of water protons present in the body. They are mainly represented by paramagnetic gadolinium complexes. Given the high toxicity of gadolinium, these contrast agents require large synthetic efforts to develop ligand cages that efficiently trap gadolinium ions. The goal is to avoid leaching of free ions and accumulation of any gadolinium containing species in the body to prevent harmful side effects such as nephrogenic system fibrosis, while trying to afford complexes with increased efficiency to reduce the dose to be injected. This concern over the long-term safety of gadolinium based contrast agents also motivates the search for less toxic (and more effective) alternatives [2,3,4]. In the last decades, so called T_2_ contrast agents have also been developed. This time the contrast in MRI arises from a decrease of the transverse relaxation time of the water protons induced by the large magnetization of the contrast agent: the local heterogeneity of the magnetic field created in the region where water molecules are diffusing leads to an acceleration of protons nuclear spins dephasing and a decrease of transverse relaxation time T_2_. These contrast agents are mainly superparamagnetic iron oxide nanoparticles (NPs) [4,5]. Based on iron, these contrast agents, the ultimate fate of which is macrophage capture and subsequent metabolism into labile iron, are potentially less toxic than gadolinium based ones. Furthermore, as for many nanomaterials, their surface may be easily modified to offer longer circulating time, multimodality [6], modulable half-life or specificity [7,8], or to offer other functionalities such as drug delivery vehicles or magnetic and photonic ablation therapies [9,10,11], which is otherwise difficult to obtain with molecular contrast agents. However, large doses still need to be injected to afford a significant contrast, which raises toxicity concerns related to iron overload [2]. To answer this issue, nanomaterials of higher magnetization and good crystallinity are needed. Among iron based nanomaterials, zerovalent iron NPs display much higher values of magnetization than the currently used iron oxides [12], which makes them attractive alternatives as T_2_ weighted MRI contrast agents. Due to their high surface reactivity towards air and water, their surface spontaneously oxidizes into a passivating thin oxide layer [13] leading to a stable iron-iron oxide (Fe@FeOx) core-shell architecture with a magnetization which remains well above that of the previously used iron oxide NPs. This system has been recognized as a promising T_2_ MRI contrast agent [14,15,16,17,18,19,20,21] but surprisingly information on its engineering is still limited. The purpose of this study is to contribute filling in this gap and report on the conditions required to afford Fe@FeOx NPs that are stable in water, on the evaluation of their efficiency in MRI and assays of their cytotoxicity.

Production of zerovalent iron NPs (NPFe) of controlled morphology is best achieved by reduction of the amido iron complex [Fe(N(SiMe_3_)_2_)_2_]_2_. Indeed, our group and others demonstrated that NPs of tunable size and shape could be obtained by adjusting simple reaction parameters [22,23,24,25,26,27]_._ The NPFe obtained are intrinsically hydrophobic due to the nature of their surface capping ligands (long chain amine). Their use in biomedical applications thus requires engineering of their surface to provide stable colloidal solutions in water. For this purpose, an amorphous silica coating is attractive. It is an efficient way to impart water solubility to hydrophobic NPs and afford colloidal aqueous solutions, which are stable for extended periods of time. Furthermore it has already been used to efficiently develop imaging contrast agents [3,28] and recent studies have reported its degradation in biological media into non toxic silicic acid [29]. Reverse emulsion methods are particularly robust to prepare amorphous silica shells of tunable thickness and have been well developed to coat magnetite NPs [30,31]. We show here that adapting this method to Fe@FeOx NPs produces NPs with water solubility and limited toxicity at the same time. Finally, PEG chains are grafted at the surface of the NPs to further improve the biocompatibility of the nanosystem [32,33]. The NPs thus obtained have been evaluated in MRI and the relationship between their magnetization, relaxivity and efficiency is discussed, as well as the effect of their surface coating and in vitro cytotoxicity by comparison to amorphous silica NPs with no Fe@FeOx core.

## 2. Results and Discussion

### 2.1. Iron NPs

**Scheme 1:**
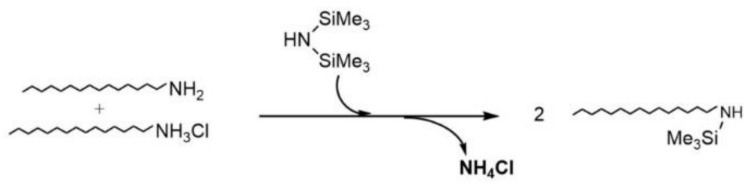
Formation of NH_4_Cl and silylated amine.

**Figure 1:**
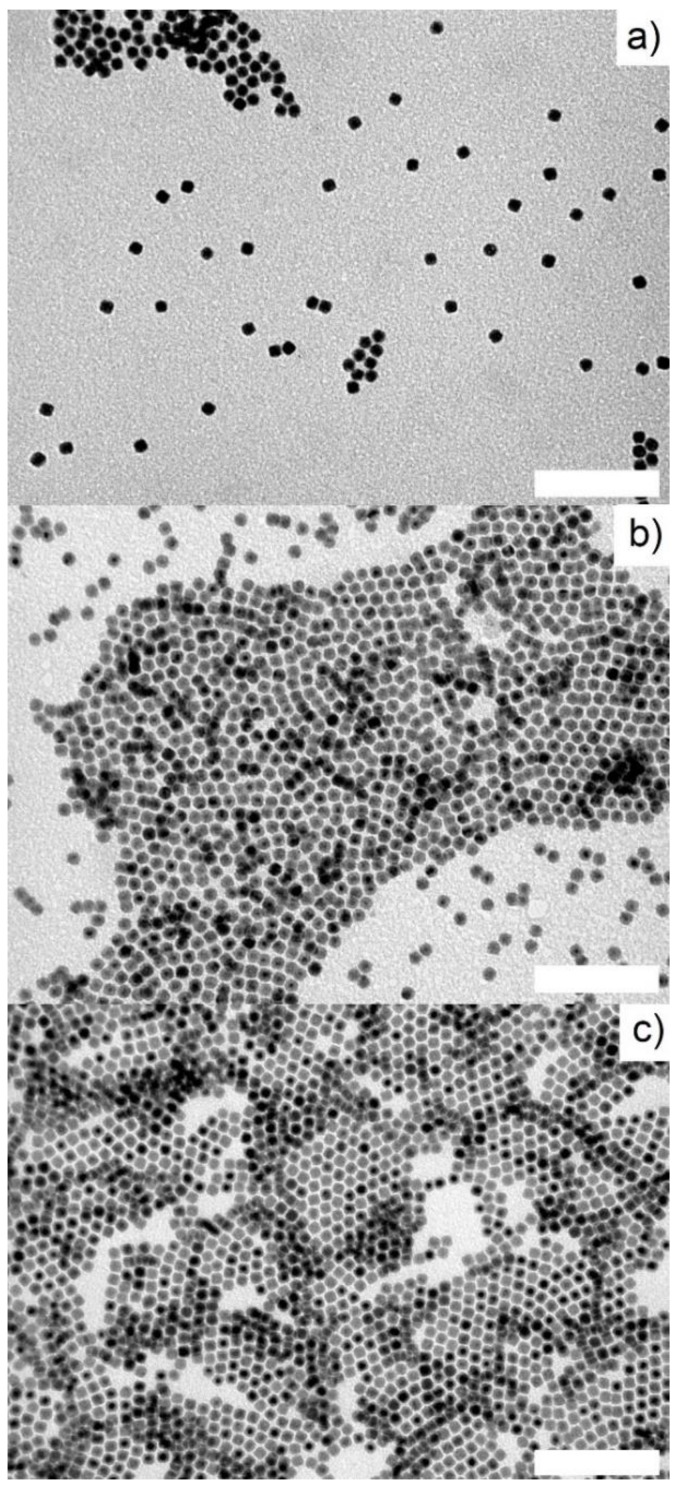
TEM images of (**a**) toluene washed zerovalent iron nanoparticle (NPFe), (**b**) ethanol washed NPFe and (**c**) oleic acid treated NPFe. Scale bar: 100 nm.

**Figure 2:**
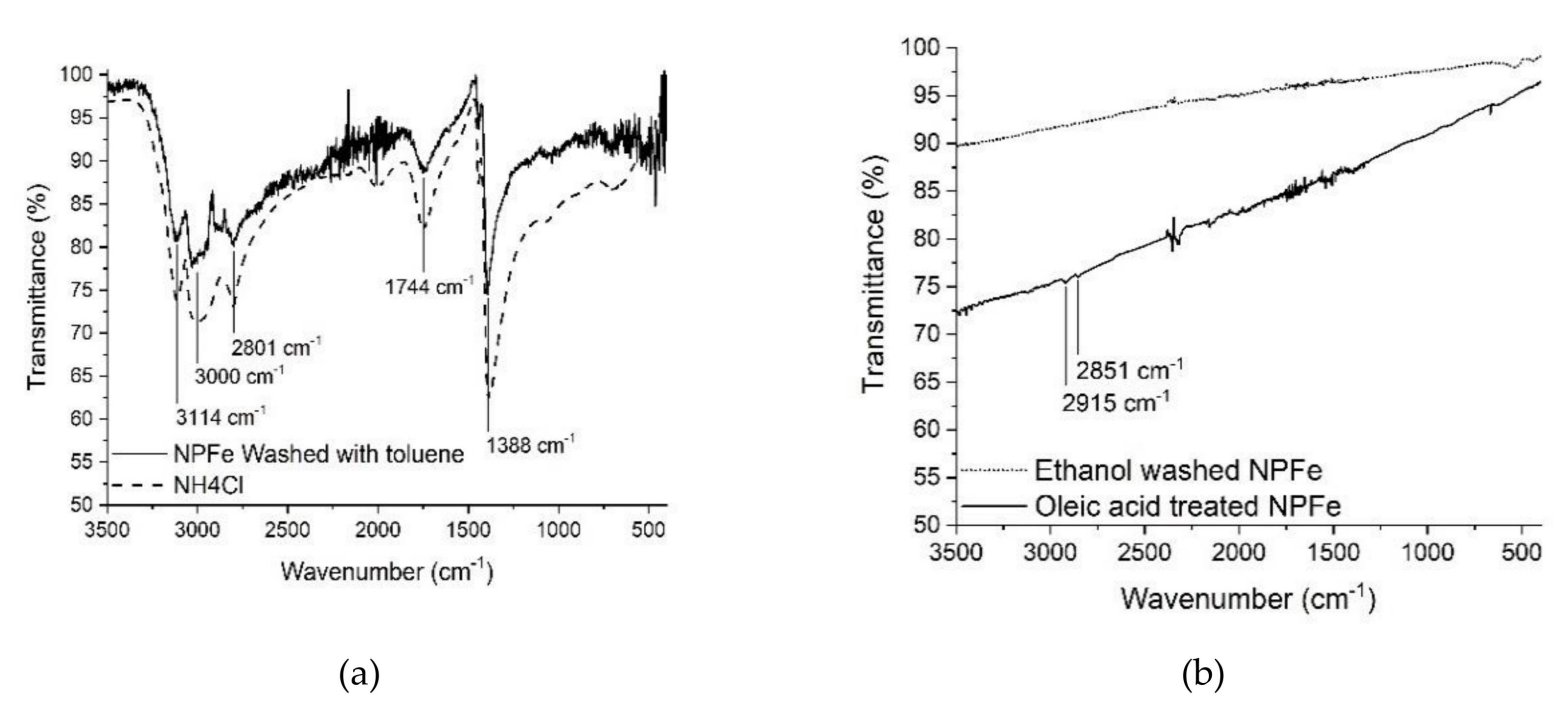
FTIR spectra of (**a**) toluene washed NPFe (solid line) in comparison with NH_4_Cl (dashed line), (**b**) ethanol washed NPFe (dotted line) and oleic acid treated NPFe (solid line).

To afford Fe@FeOx NPs comprising a passivating FeOx shell we chose to start from zerovalent iron NPs. Indeed, the spontaneous oxidation of zerovalent iron NPs in air has already been shown by our group and others to lead to the target core-shell nanomaterial. We selected a versatile synthesis method that provides NPs of clean surface state for their increased reactivity towards dioxygen. Thus iron NPs were synthesized using [Fe(N(SiMe_3_)_2_)_2_]_2_ as a precursor and a mixture of HDA/HDA.HCl in mesitylene, a method reported to lead to hexamethyldisilazane and hexadecylamine as the only by-products [27]. This method also yielded NPs of high crystallinity that were expected to give better results in MRI than amorphous ones [16,34]. After 65 h at 150 °C, a black powder was obtained by magnetic separation, which was washed with toluene. A dispersion of this powder in toluene was drop casted on a carbon coated copper grid and analyzed by TEM (Figure 1a). The micrograph showed NPs with an average size of 9.8 ± 1.6 nm (Figure S1a). This size is well adapted for the application envisaged. It is large enough to avoid complete corrosion of the NPs upon air exposure [13] and to ensure large enough magnetization after oxidation so that the material can be easily purified and handled, and it is small enough to prevent dipolar magnetic coupling in the final material (vide infra). The powder was further analyzed by IR spectroscopy (ATR mode; Figure 2a and Figure S2). Surprisingly, the typical signatures of HDA and HDA.HCl could not be observed on the spectrum suggesting that only traces of these compounds were trapped in the powder. Rather, the large bands in the region 3114–2801 cm^−1^, 1744 cm^−1^, and 1388 cm^−1^ corresponded to νN-H vibrations of NH_4_Cl [35,36], as already observed by Gharbi et al. [37]. As in this previous work, NH_4_Cl most probably arose from a side reaction between HDA.HCl and HMDS (Scheme 1). The fate of HDA and HDA.HCl was further investigated by studying the supernatants recovered during the purification process after magnetically collecting the NPs. The IR spectrum of the impurities extracted with toluene displayed νC-H stretching bands at 2840 cm^−1^ and 2912 cm^−1^ indicative of the presence of long alkyl chains but the absence of the νN-H vibrations of HDA and HDA.HCl expected at 3330cm^−1^, 3260cm^−1^, and 3170cm^−1^ for HDA and around 3000 cm^−1^ for HDA.HCl confirmed the modification of the ligands (Figure S3a). A new band at 1670 cm^−1^ indicated the formation of an imine as a result of the reduction of the iron precursor by HDA as already reported by Meffre et al. [27]. Furthermore, a new νSi-C vibration was observed at 1248 cm^−1^, which could not be attributed to hexamethyldisilazane given the absence of the characteristic νN-H band (expected at 1174 cm^−1^). This new νSi-C vibration was then attributed to N-trimethylsilylhexadecylamine based on the work by Gharbi et al. [37] who showed that a monosilylamine was formed in these conditions (Scheme 1). Transformation of HDA.HCl into toluene soluble N-trimethylsilylhexadecylamine greatly facilitated the purification of the NPs. Removal of NH_4_Cl and remaining traces of HDA.HCl could then be performed by washing the powder with ethanol, as confirmed by IR analysis of the magnetic powder, which displayed only a flat spectrum at the end of the purification process [38] (Figure 2b).

At this step, the NPs were not soluble in any solvent (organic or aqueous) but a TEM grid could still be prepared from dispersion in toluene. The average size of the NPs was unchanged with a 9.7 ± 1 nm size (Figure 1b and Figure S1b).

The surface of the NPs was then functionalized by oleic acid to afford colloidal solutions in organic solvents without any significant change of their average diameter as determined from the TEM images collected at this stage (final average size of 9.3 ± 1 nm, Figure 1c and Figure S1c). The IR spectrum (ATR mode) of this nanomaterial displayed signals at 2915 cm^−1^ and 2851 cm^−1^ that could be attributed to ν(CH) vibrations in alkyl chains in agreement with surface grafting of oleic acid (Figure 2b). Their low intensity is related to the limited quantity of oleic acid (close to a monolayer) in comparison to the large iron content in the material (here above 80%w). 

### 2.2. Silica Coated Fe@FeOx NPs

**Figure 3:**
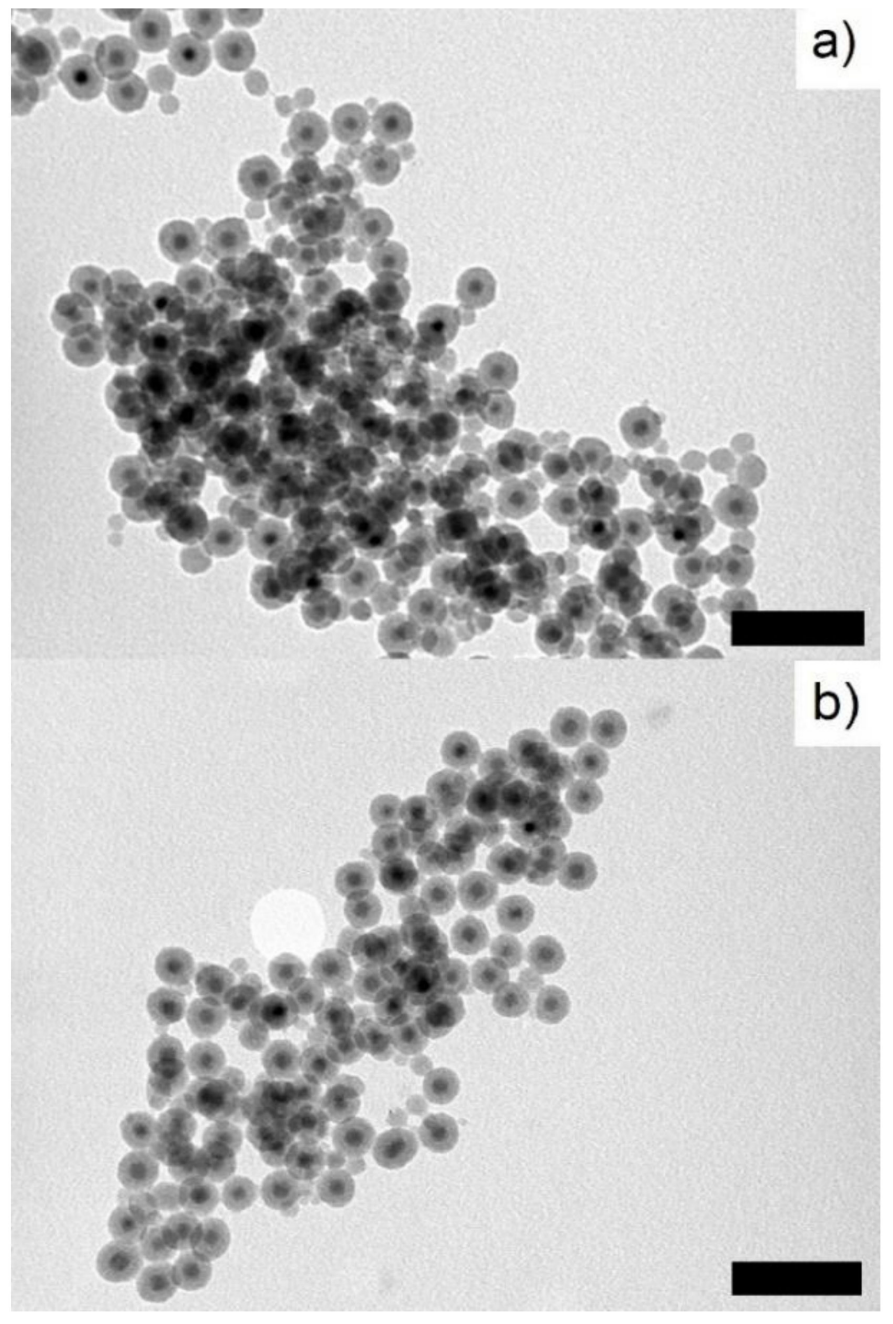
TEM micrographs of (**a**) NPFe@FeOx@SiO_2_ and (**b**) NPFe@FeOx@SiO_2_-PEG. Scale bar: 100 nm.

Coating of the NPs by an amorphous silica shell was carried out in reverse micellar medium according to the work of Ding et al. [30]. Igepal reverse micelles were formed in cyclohexane/water medium. Dispersion of the NPs in the oil phase was eased out by the presence of oleic acid at their surface. Then the solution was exposed to air to generate a thin iron oxide shell. Formation of the oxide has been reported elsewhere [39,40]. Whether it consisted of maghemite, magnetite or a gradient of both was not investigated in this work but it is generally admitted that magnetite is the main component of the oxide shell close to the residual iron core while the interface with air and water mostly consists in maghemite [13,41,42,43]. This oxidation step had important falls out: first it stabilized the iron core against further oxidation (vide infra) and allowed its easy handling, then it provided OH functional groups when in contact with water which allowed grafting of the silica layer otherwise impossible to achieve for clean metal surfaces [44], and last it limited the dipolar magnetic coupling between NPs as a result of the decrease of their neat magnetization. This point was important to avoid the formation of chains of NPs and allowed reaching a homogeneous dispersion of the NPs in the oil phase, a key point to get only one Fe@FeOx NP inside each silica shell. A careful adjustment of the quantity of NPs versus surfactant and TEOS limited the formation of silica NPs (NPSiO_2_) with no magnetic core. After removal of igepal by extensive centrifugation/redispersion washing steps, TEM grids were prepared by drop casting a water dispersion of the NPs on a carbon coated copper grid. The micrographs displayed only a limited number of NPs without any core but mostly NPs with a dark core surrounded by a shell of lighter contrast (Figure 3a) [45]. Statistical analysis of the TEM images evidenced 10.7 ± 2 nm thick silica shells with Fe@FeOx cores of 10.2 ± 2 nm (Figure S4), yielding global structures of 32 ± 3.2 nm average diameter.

It is noteworthy that the NPs could still be manipulated with a simple magnet despite partial oxidation of the iron core and did not display any strong magnetic dipolar couplings as evidenced by the absence of chain-like arrangements on the TEM grid. The absence of magnetically induced aggregation is important in view of biological applications as they would otherwise induce the clotting of the blood vessels. Dynamic light scattering (DLS) studies were performed to further assess the stability of the aqueous solutions. Results are reported on Table 1. Freshly prepared dispersions of silica coated NPs reproducibly displayed an average hydrodynamic diameter of around 100 nm but the NPs slowly aggregated over time yielding to two populations in the intensity weighted distribution of sizes after 2 months in water (Figure S7a). Zeta potential analysis showed no variation of their surface charges at physiological pH nor of their isoelectric point over this period (Figure S7b). Obviously, even at zeta potential values of circa –40 mV, surface charges were not sufficient for the electrostatic repulsion to overcome the combined strong Van der Waals interactions setting in at this size range and gravity forces. We thus proceeded to graft PEG chains at the surface of the NPs to try and impart a better stability to the system and afford nanomaterials of improved biocompatibility [32].

### 2.3. Surface Functionnalization

PEGylation of the NPFe@FeOx@SiO_2_ was achieved by reacting 5 kDa PEG chains, previously modified to accommodate a triethoxysilane function, with NPFe@FeOx@SiO_2_ in a 2/1 *v/v* water/ethanol mixture for 48 h at 50 °C. Condensation of the triethoxysilane end group onto the silica surface was catalyzed by ammonia. After extensive purification, the dark brown product obtained was analyzed by NMR, IR spectroscopy, TEM, and DLS, and the data were compared to those recorded from NPFe@FeOx@SiO_2_. TEM analysis of the sample (Figure 3b and Figure S5) showed that the reaction yielded a nanosystem with an unchanged size for the Fe@FeOx core (9.7 ± 2.7 nm) but a thinner silica shell (8.5 ± 1.7 nm). The shrinking of the shell was probably a consequence of the supplementary 48 hours of reaction in a basic environment at 50 °C, thus continuing the condensation of the silica on itself. This has expected advantages: first it might impart an added protection to the magnetic core, second the silica shell should present an added resistance towards the biological medium, last the distance between the core and the water protons being reduced, the relaxivity of the NPs should be better [46]. DLS study showed no significant increase in the size of the NPs upon grafting of the PEG chains at their surface, which we could relate to a low grafting density: here 1 PEG/32 nm² (Table 1 and Figure S8a). Indeed in these conditions PEG chains were expected to adopt a mushroom configuration (up until 1 PEG/24.4 nm²) [47] rather than fully extend their chains into water. After two months of settling in water, the hydrodynamic diameter increased from 110 to 200 nm. The isoelectric point was not modified by the PEGylation, in agreement with the low grafting density. Similarly, the surface potential between pH 5 and pH 10 remained below −30 mV for all analyzed samples even after two months of retention in MilliQ water (Figure S8b).

**Figure 4:**
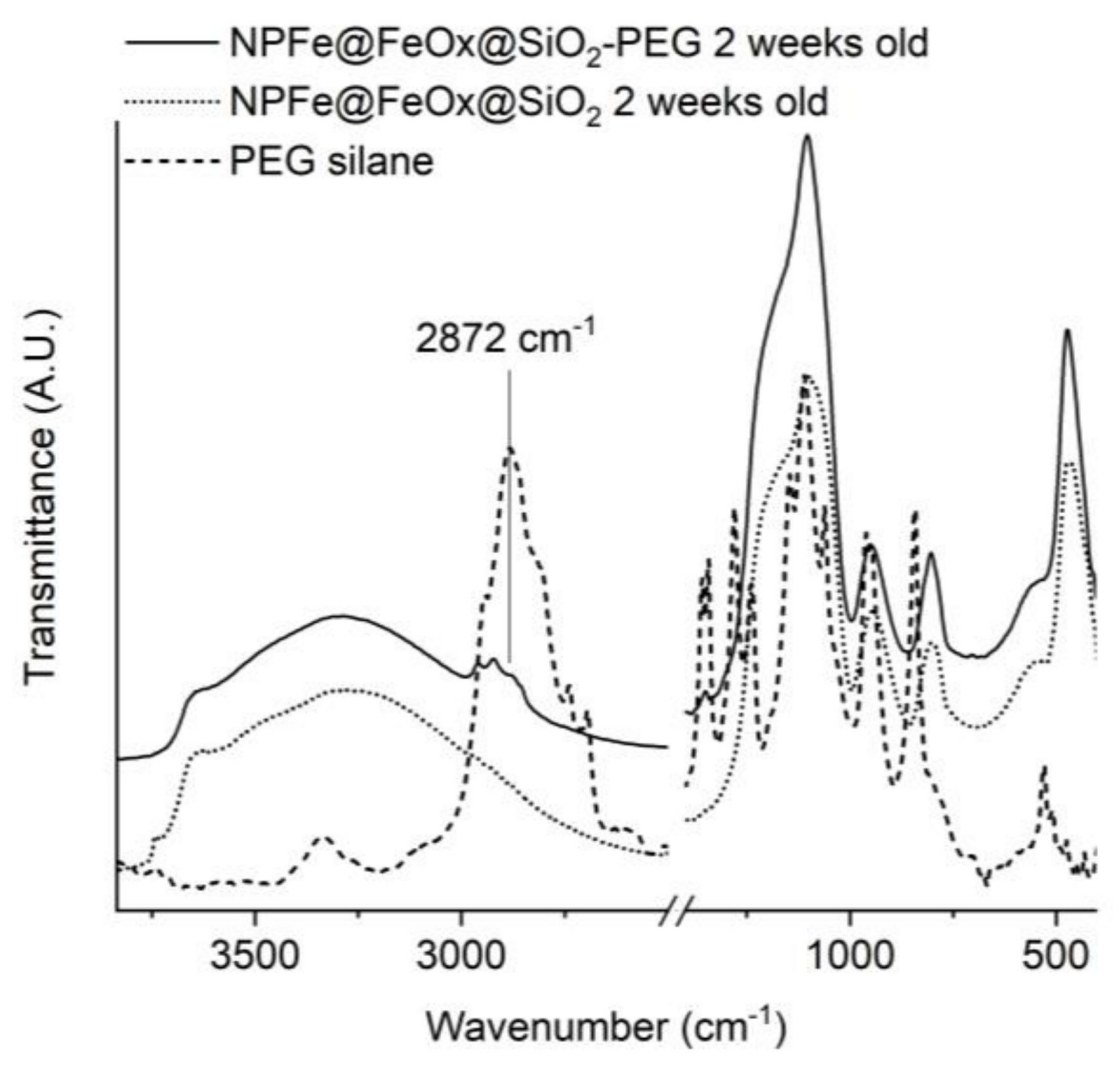
DRIFT IR spectra of NPFe@FeOx@SiO_2_ (dotted line), NPFe@FeOx@SiO_2_-PEG (solid line), and PEG-silane (dashed line).

PEGylation was followed by IR spectroscopy. Spectra were recorded in diffuse reflectance mode (DRIFT) to increase the signal over noise ratio. By comparison between the spectra of the PEG, NPFe@FeOx@SiO_2_ and NPFe@FeOx@SiO_2_-PEG, the new vibration bands observed at circa 2872 cm^−1^ in the DRIFT spectra of NPFe@FeOx@SiO_2_-PEG (Figure 4) could be attributed to the C–H stretching bands of the PEG chains and suggested the success of the PEGylation of NPFe@FeOx@SiO_2_.

**Figure 5:**
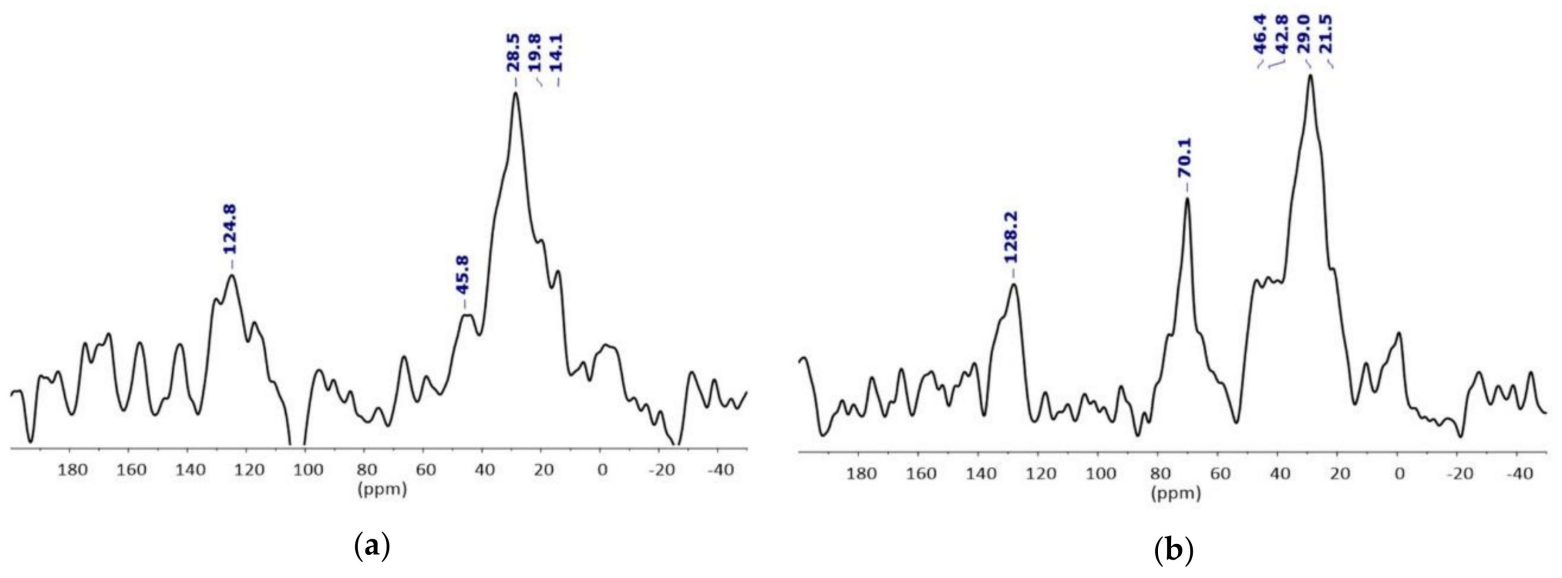
^13^C MAS NMR spectra (recovery delay of 0.5 s) of (**a**) NPFe@FeOx@SiO_2_ and (**b**) NPFe@FeOx@SiO_2_-PEG.

This was further confirmed by a comparison of the ^13^C magic angle spinning (MAS) NMR spectra of the NPs before and after PEGylation (Figure 5). Due to the paramagnetism of the NP, the ^13^C relaxations were fast and the ^13^C MAS was recorded with a short recovery delay of 0.5 s. The prominent features of the NPFe@FeOx@SiO_2_ spectrum could be attributed to the oleic acid (14–46 ppm: C of aliphatic chain, 124–129 ppm: C of the C=C bond) bounded to the magnetic core surface. In comparison, the ^13^C MAS spectrum of NPFe@FeOx@SiO_2_-PEG displayed a new peak at 70.1 ppm, which was assigned to ethoxy groups from the PEG chain. As the relaxation times of the different nuclei present in the material depend on their distance to the paramagnetic core, the observation of signals associated to the surface bound oleic acid was facilitated.

### 2.4. Magnetic and Relaxometric Properties

**Figure 6:**
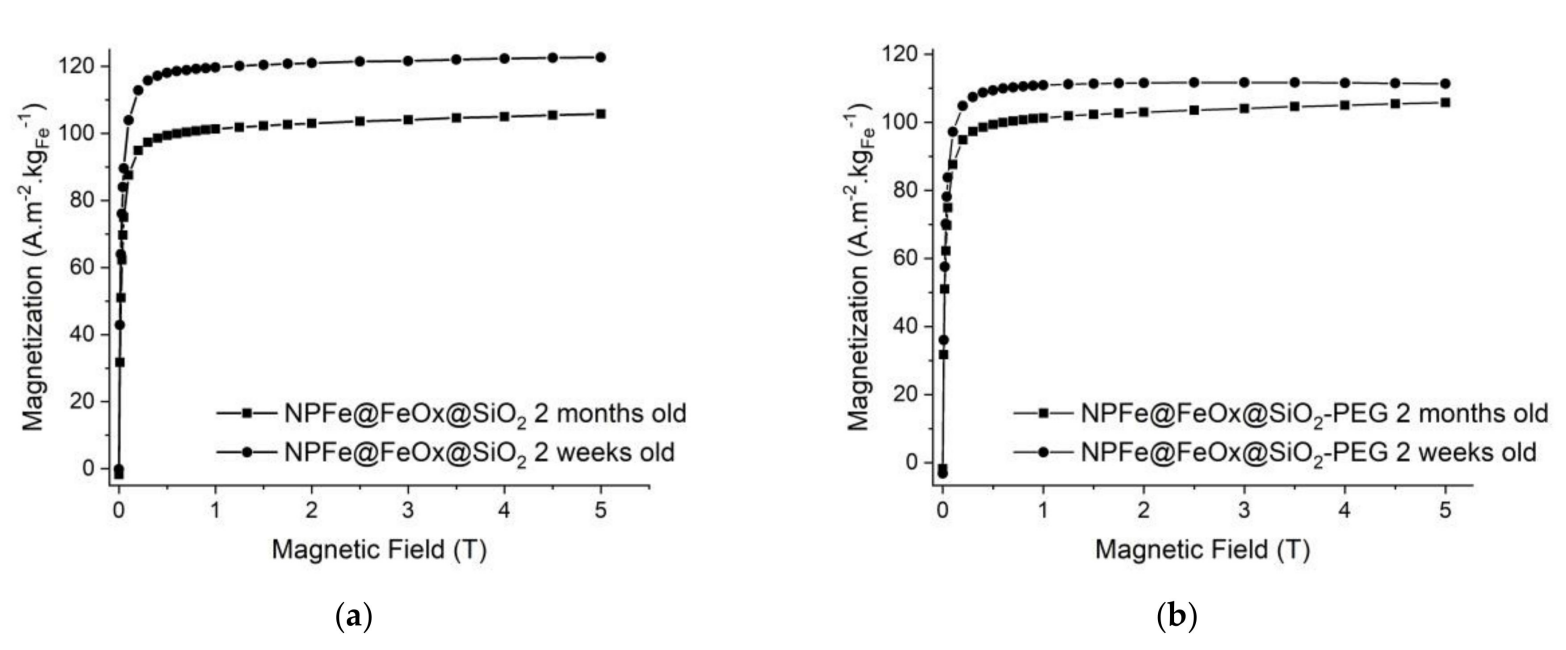
Magnetization curves at 37 °C of (**a**) NPFe@FeOx@SiO_2_ and (**b**) NPFe@FeOx@SiO_2_ PEG after 2 weeks and 2 months of retention in water.

The magnetic properties of the samples were studied at 37 °C (Figure 6). It is noteworthy that in both cases, the magnetization was already very close to its saturation state at 1 T, the lowest value of the magnetic field used for the imaging of phantoms (vide infra). It is noteworthy that the magnetic field levels of the current MRI are around 1.5–3 T. As expected given the surface oxidation of the NPs, saturation magnetization values of both samples lied between the one for bulk iron and those commonly reported for iron oxide NPs (i.e., below 90 A.m^2^ kg^−1^). Importantly these values remained higher than the one reported for pure oxides developed for biomedical applications among which RESOVIST® (M_s_ = 95 A.m^2^ kg_Fe_^−1^). 

The saturation magnetization of NPFe@FeOx@SiO_2_ was 123 A.m². kg_Fe_^−1^ (measured at 5 T) and decreased by 20% after 2 months in MilliQ water in aerobic conditions.

For the NPFe@FeOx@SiO_2_-PEG sample, the magnetization saturated at a somewhat lower value, 110 A.m^2^. kg_Fe_^−1^ at 5 T, which could be explained by the extended period in basic medium during the grafting of the PEG derivative favoring the formation of the oxide. The magnetization decreased to 105 A.m^2^. kg_Fe_^−1^ after 2 months in water (i.e., by less than 5%).

The thickness of the oxide layer could be estimated around 0.5 nm (80% of the magnetic core volume) and 1.1 nm (88% of the magnetic core volume) for NPFe@FeOx@SiO_2_ and NPFe@FeOx@SiO_2_-PEG sample respectively based on the work by Lacroix et al. [16] and in agreement with previously reported data [48]. After 2 months under water in aerobic conditions, the magnetization decrease is associated to an increase of the shell thickness to respectively 1.1 nm (88% of the magnetic core volume) and 1.8 nm (93% of the magnetic core volume) for NPFe@FeOx@SiO_2_ and NPFe@FeOx@SiO_2_-PEG samples respectively.

**Figure 7:**
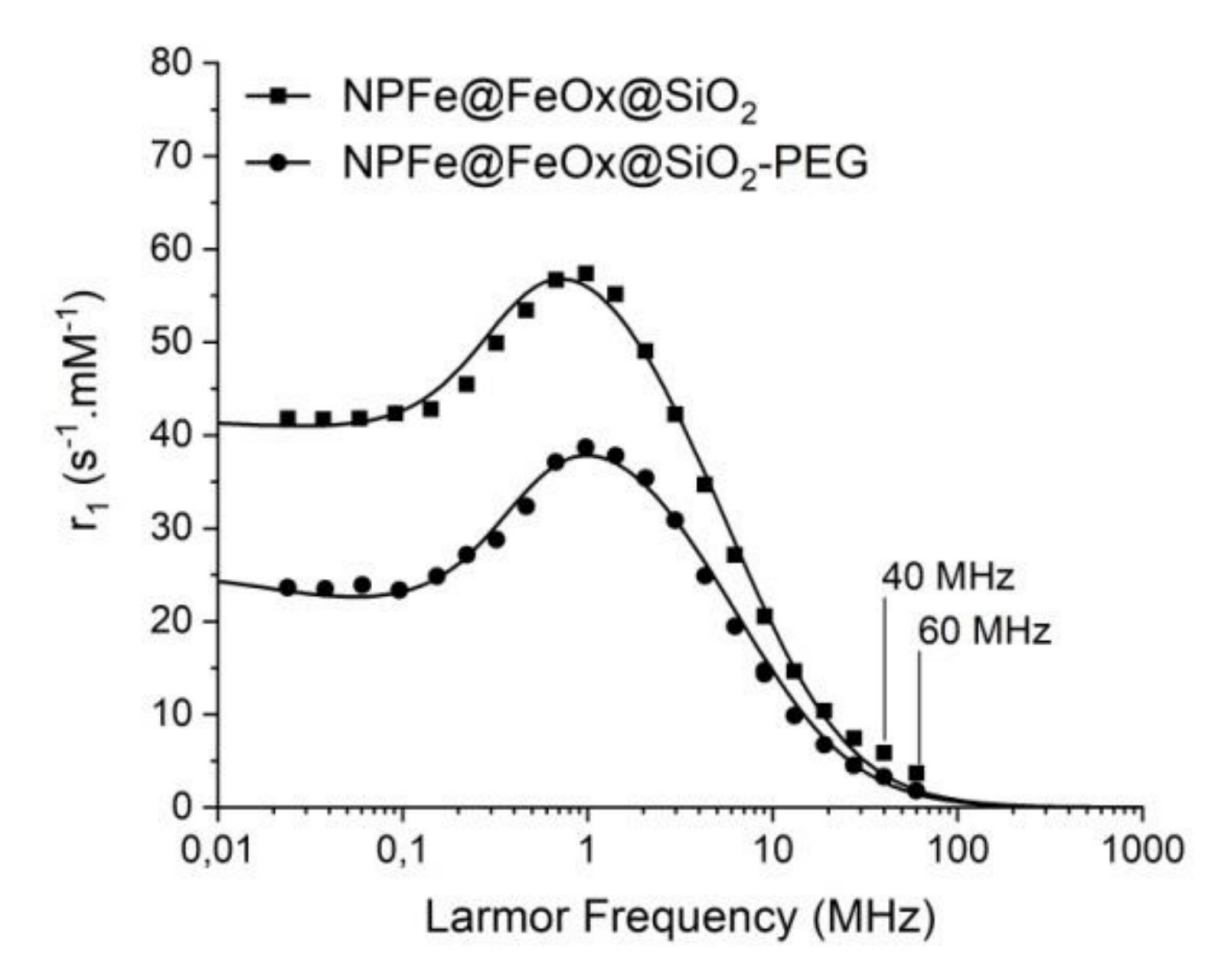
Fitted NMRD profiles of NPFe@FeOx@SiO_2_ and NPFe@@FeOx@SiO_2_-PEG.

Longitudinal relaxation NMRD profiles were recorded at 37 °C for both nanomaterials (Figure 7). They displayed the characteristic shape expected from superparamagnetic NPs with a low field plateau, a large peak around 1 MHz and strong decrease to zero at high frequencies [49]. A fit of the data (Figure 7 and Table 2) allowed estimating the size and saturation magnetization (Ms) of the active material, as well as Néel relaxation time (τ_Néel_), the diffusion coefficient (D) being fixed to that of water at 37 °C (D = 3.01 10^−5^ cm^2^/s). Independent of PEG grafting, the average diameter was estimated around 28 nm, which was in good agreement with the size determined by TEM analysis (e.g., for the NPFe@FeOx@SiO_2_-PEG sample, the Fe@FeOx core (9.7 ± 2.7 nm) plus silica shell (8.5 ± 1.7 nm) corresponded to an overall diameter of around 27 nm), showing that the silica coating should be envisaged as impermeable to water at the time scale of the measurement [50]. Moreover the results reported herein showed that the PEG coating did not alter the diffusion of water in the vicinity of the NPs and confirmed that aggregation of the NPs was negligible. By considering the silica coating as impermeable to water, the magnetization deduced from the fit compared well to the one measured by SQuID analysis, being slightly lower after PEG grafting as discussed above. Indeed, as described by Pinho et al. [50], a correlation can be obtained between both measurements by applying a correction factor to the magnetization obtained by SQuID, which takes into account the diameters of the coating and of the crystal. Magnetizations of 21 A.m^2^.kg_MM_^−1^ (MM = magnetic material, i.e., iron core plus coating) and 18 A.m^2^.kg_MM_^−1^ can be estimated for Fe@FeOx@SiO_2_ and Fe@FeOx@SiO_2_-PEG nanoparticles respectively, in accordance with the values obtained by relaxometry. Measurements at low field pointed to a lower anisotropy energy for the PEGylated system.

Assessment of effect of the NPs on the relaxation times of water protons was investigated at 20 MHz and 60 MHz (respectively 0.47 and 1.5 T) at 37 °C. Relaxivities are reported on Table 3, as well as those for RESOVIST® (presently the only commercially available iron oxide based MRI contrast agent) and DOTAREM® (currently used to increase the contrast in T_1_ weighted MRI), which were measured in the same conditions for the sake of comparison. Silica coated NPs and RESOVIST® displayed similar transverse relaxivities (r_2_) values, while the relaxivity of the NPs was enhanced after functionalization by PEG. As the magnetization of the NPs slightly decreased after 2 months in water the observed decrease of their r_2_ values was expected, as already reported by Masoudi et al. [21]. 

In agreement with the NMRD profiles, no significant difference could be made between the longitudinal relaxivities (r_1_) of the NPs, PEGylated or not. Interestingly these values were systematically lower than those measured for RESOVIST®. Consequently the r_2_/r_1_ ratios displayed by NPFe@FeOx@SiO_2_ and NPFe@FeOx@SiO_2_-PEG were higher compared to RESOVIST®, especially when working at 1.5T. A stronger darkening, hence better contrast in the images, was then expected.

There are few data that allow direct comparison with the results reported herein. Indeed, the value of the relaxivity depends on many factors [3] such as magnetization of course, but also magnetic anisotropy energy (both parameters that can be influenced by the nature of the molecules coordinating at the surface of the magnetic core), size, thickness of the coating and permeability to water molecules, aggregation, and temperature to cite a few. Longitudinal relaxation rates (r_1_) recorded at 20 MHz compared well with those reported by Yoon et al. [19] for DMSA capped Fe@FeOx NPs of larger magnetic core (16 nm) and thus larger saturation magnetization (140 A.m^2^.kg_Fe_^−1^) but much smaller hydrodynamic diameter (<45 nm) while they reported larger transverse relaxivities leading to better r_2_/r_1_ values (around 30). Another comparison can be made with the work of Khurshid et al. [51] who reported r_2_ values around 180 mM^−1^.s^−1^ at 60 MHz for PEGsiloxane coated Fe@FeOx NPs with comparable sizes and surface state (as far as the magnetic core is concerned). However the longitudinal relaxivity was not studied in this work and no clear conclusion on the contrast expected in MRI can be drawn. CG Hadjipanayis [17] also reported lower r_2_ values for Fe@FeOx NPs of the same size with a thin (2–3 nm) carboxyPEG surface coating but amorphous iron core, emphasizing the importance of the crystallinity of the core nanomaterial, as reported by Lacroix et al. [16] and Herman et al. [34], and further evidenced in the present work. 

**Figure 8:**
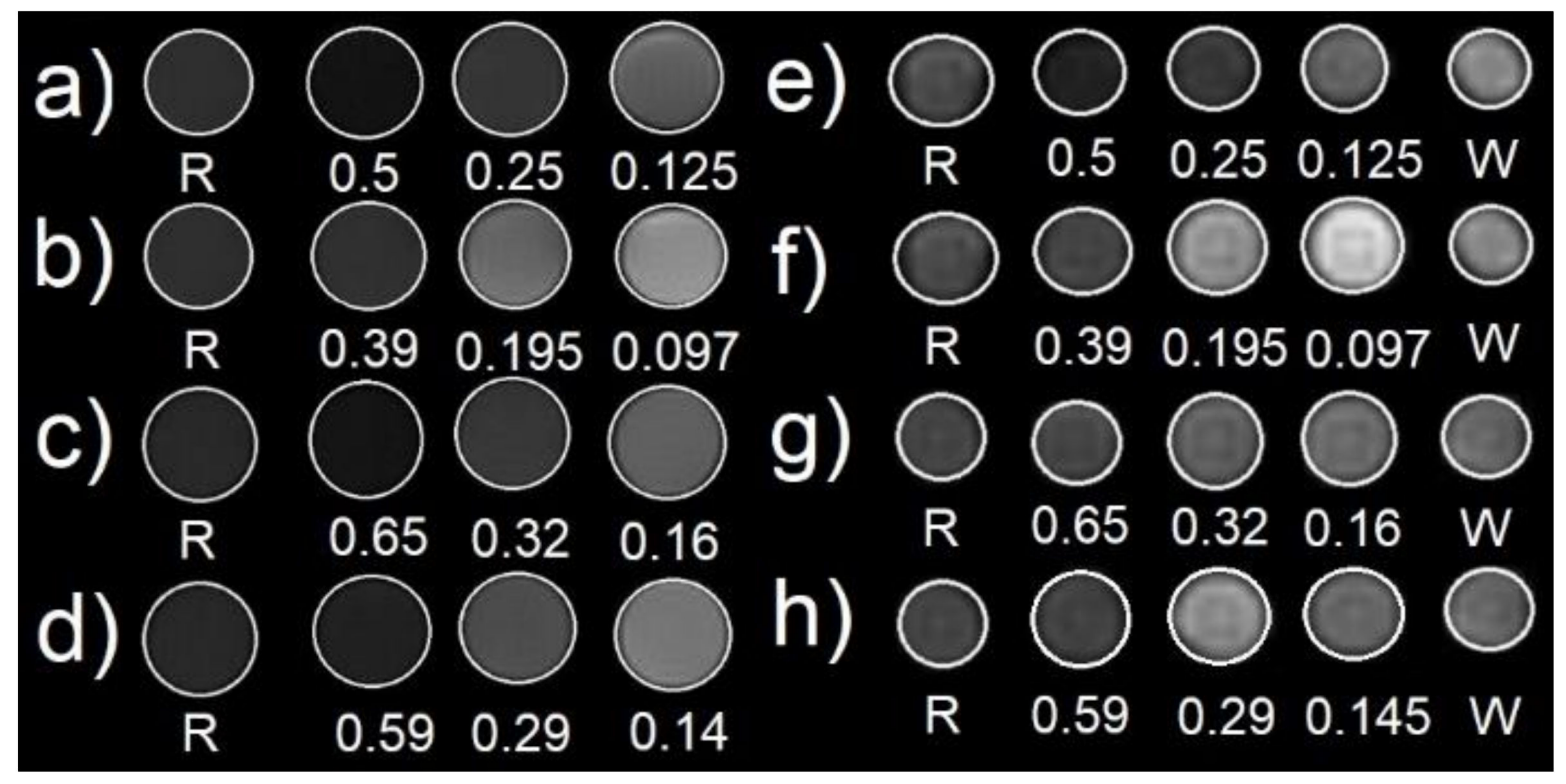
T_2_ magnetic resonance imaging (MRI) phantoms images acquired at 9.4 T (TR = 5000 ms, TE = 8 ms): (**a**) NPFe@FeOx@SiO_2_-PEG 2 months old, (**b**) NPFe@FeOx@SiO_2_-PEG 2 weeks old, (**c**) NPFe@FeOx@SiO_2_ 2 months old, (**d**) NPFe@FeOx@SiO_2_ 2 weeks old and at 1 T (TR = 5000 ms, TE = 8 ms), (**e**) NPFe@FeOx@SiO_2_-PEG 2 months old, (**f**) NPFe@FeOx@SiO_2_-PEG 2 weeks old, (**g**) NPFe@FeOx@SiO_2_ 2 months old, and (**h**) NPFe@FeOx@SiO_2_ 2 weeks old. Concentrations are in mmol.L^−1^, R stands for RESOVIST® (0.5mM) and W stands for water.

To confirm the expected darkening effect from the nanomaterials under study, MRI images of phantoms were acquired at 1 T and 9.4 T at 37 °C according to T_1_ and T_2_ weighted sequences in a concentration range 0.1–0.7 mmol. L−1. As expected, the NPs induced no contrast in T1 images (Figure S9) but were active in T2 weighted sequences (Figure 8). The NPFe@FeOx@SiO_2_, PEGylated or not, were once again compared to RESOVIST® introduced at a concentration in iron of 0.5 mmol. L^−1^, typical for clinical use. Comparison between the images clearly proved that, at identical concentration, NPFe@FeOx@SiO_2_ and NPFe@FeOx@SiO_2_-PEG both induced a better darkening effect than RESOVIST® even at high field [52]. It suggests that this contrast agent could be used to get well resolved images and to ease the detection of very small tumors, a key for successful treatment of cancers. Furthermore, the images recorded at 1 T in the presence of the nanosystems displayed an enhanced darkening effect compared to the one recorded with RESOVIST® already at lower concentrations, which suggests that the dose to be injected to get relevant images for clinical applications could be reduced. From these images r_2_ values at 9.4 T could be estimated (Table 3).

The T_2_ NMRD profile was not determined for technical reasons but, as expected [49], one could observe that r_2_ increased with field strength, reaching values around 240 mM^−1^.s^−1^ at 9.4 T for the PEGylated samples. In comparison, McGrath et al. [53] reported r_2_ values slightly below 230 mM^−1^.s^−1^ at room temperature for Fe@FeOx NPs with comparable core size (11 ± 2 nm) and saturation magnetization (103 A.m^2^.kgFe^−1^), but larger hydrodynamic diameter (180 nm) due to presence of phosphonate-grafted polyelectrolytes at their surface.

### 2.5. Cytotoxicity Assessment

**Figure 9:**
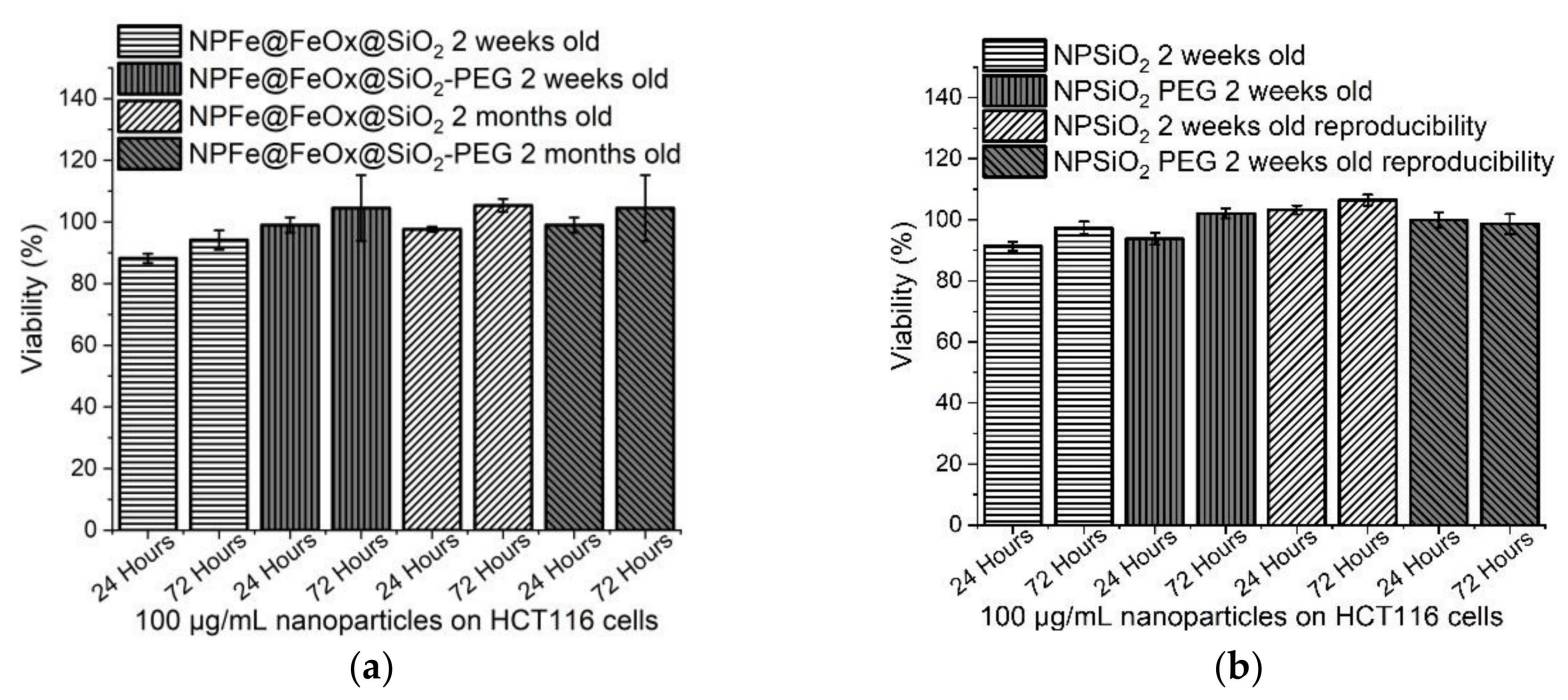
Cytotoxicity assays of (**a**) NPFe@FeOx@SiO_2_ (100 µg/mL) and NPFe@FeOx@SiO_2_-PEG (100 µg/mL) and (**b**) NPSiO_2_ (100 µg/mL) and NPSiO_2_-PEG (100 µg/mL) on HCT116 cells after 24 hours and 72 hours of incubation at 37 °C.

**Figure 10:**
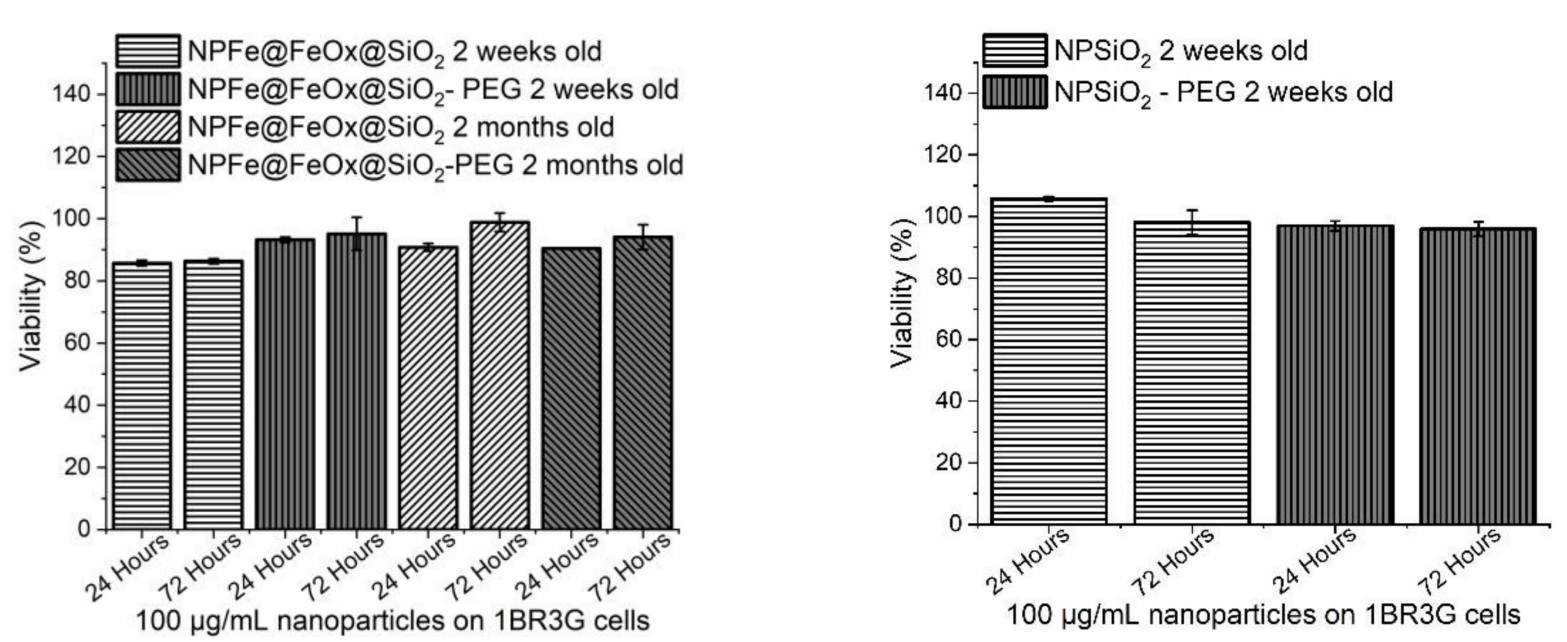
Cytotoxicity assays of (**a**) NPFe@FeOx@SiO_2_ (100 µg/mL) and NPFe@FeOx@SiO_2_-PEG (100 µg/mL) and (**b**) NPSiO_2_ (100 µg/mL) and NPSiO_2_-PEG (100 µg/mL) on 1BR3G cells after 24 hours and 72 hours of incubation at 37 °C.

**Figure 11:**
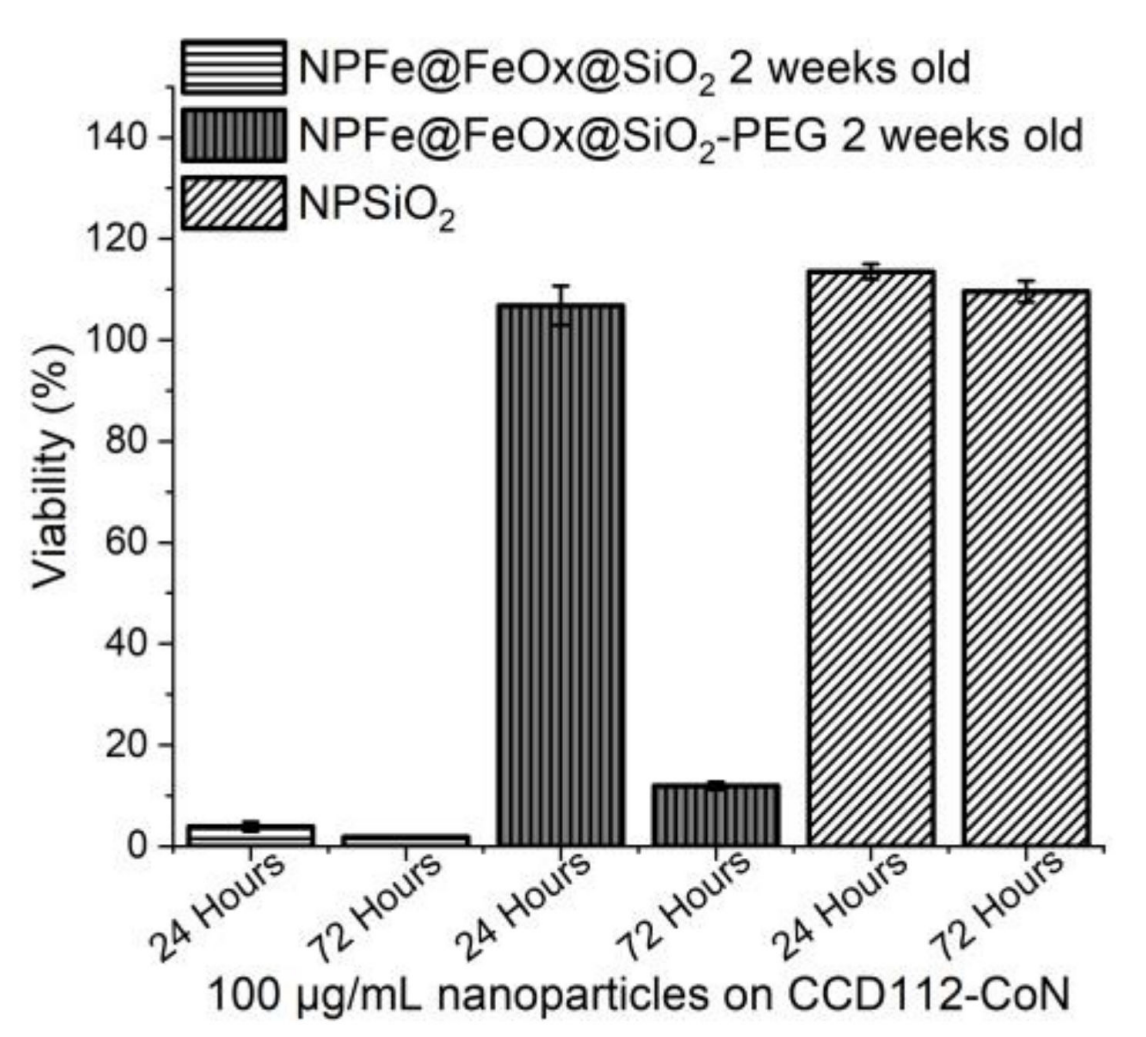
Cytotoxicity assays of NPFe@FeOx@SiO_2_ (100 µg/mL), NPFe@FeOx@SiO_2_-PEG (100 µg/mL), and NPSiO_2_ (100 µg/mL) and on CCD112-CoN cells after 24 hours and 72 hours of incubation at 37 °C.

Cytotoxicity assays have been performed using a human colon cancer cell line, HCT116 (Figure 9 and Figure S11), and two non tumoral fibroblast cell lines: 1BR3G (human skin fibroblasts, Figure 10 and Figure S12) and CCD112-CoN (normal colon fibroblasts, Figure 11 and Figure S13), working with a concentration of 100 µg/mL of NPs (i.e., an average of 0.5 mM of iron). Comparison was made with pure silica NPs synthesized according to the same procedure, and displaying the same surface state in order to try and evidence any possible adverse effect of the magnetic core (synthesis and characterization of the NPSiO_2_ and NPSiO_2_-PEG samples are reported in the supporting material). All the results were reproducibly observed.

For all samples tested, no significant effect on cell viability could be observed for HCT116 cell line (Figure 9) even after 72 h of incubation at the highest concentration tested (100 µg of NPs/mL). The minor differences observed all fell within the error bar of the assays. So, no effect of the presence, or not, of the iron core, and of the surface state of the NPs (PEGylated or not) could be observed on this cell line.

Cytotoxicity assays carried out on 1BR3G cell line showed only a very limited toxicity of NPFe@FeOx@SiO_2_ with a cell viability over 80% even after 72 h of incubation (Figure 10). PEGylated NPs showed a slightly increased viability of the cells in the short term, but after 2 months in water, no toxicity difference between the PEGylated and non PEGylated NPs could be observed on this cell line.

On the contrary, assays on the more sensitive CCD112-CoN cells (Figure 11) showed a high toxicity from NPFe@FeOx@SiO_2_, with a viability of the cells decreasing below 10% for NPFe@FeOx@SiO_2_ at 100 µg/mL while 100% cell viability was retained for NPFe@FeOx@SiO_2_-PEG at 24 hours of incubation. Still, the viability of the cells decreased to 15% at 72 hours of incubation for this PEGylated sample. In comparison, pure silica NPs had no toxicity on this cell line. This clearly showed the impact of the iron core and the benefit conveyed by PEG grafting. 

**Figure 12:**
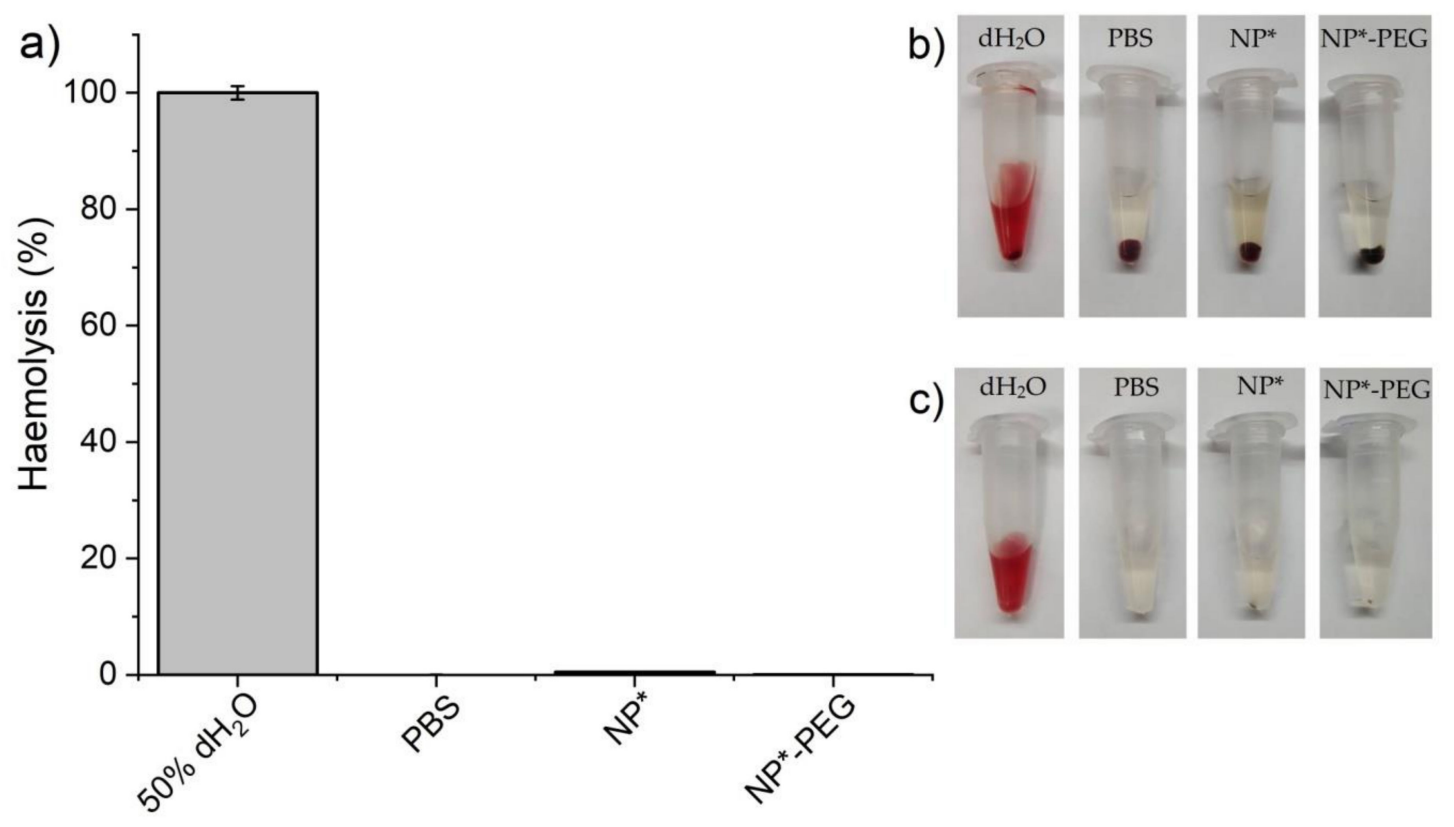
In vitro hemolysis test results for diluted human blood exposed to NPs. (**a**) Hemolysis (%) after 4 h incubation. Values represent mean ± SD from experiments performed in triplicate. (**b**) Image of the microtubes after centrifugation (800× *g* for 15 min). (**c**) Image of the microtubes after discarding the remaining nanoparticles by centrifugation (16,000× *g* for 15 min) to avoid interference with absorbance measurements. NPs*: NPFe@FeOx@SiO_2._

Last, hemocompatibility of the NPs was evaluated in vitro. Only the positive control showed visible hemolytic activity after centrifugation (800× *g* for 15 min) while all the human red blood cells (HRBCs) precipitated undamaged after incubation with the nanoparticles (Figure 12b). The remaining nanoparticles were discarded by centrifugation (16,000× *g* for 15 min) to avoid interference with absorbance measurements (Figure 12c). The hemolytic activity recorded was less than 1% for all the nanoparticles in the studied conditions (Table S1 and Figure 12a) showing that NPFe@FeOx@SiO_2_ and NPFe@FeOx@SiO_2_-PEG nanoparticles did not affect HRBCs.

## 3. Materials and Methods 

Synthesis of iron NPs (FeNP) were performed under argon atmosphere by using Fisher–Porter tubes, a glovebox and argon/vacuum lines. Mesitylene (+99%, Acros), toluene (99%, VWR Prolabo), methanol (96% STREM), acetic acid (98% Aldrich), and ethanol (99.9% Aldrich) were dried according to classical procedures. These solvents were distilled and degassed prior use. Iron bistrimethylsilylamide dimer, [Fe(N(SiMe_3_)_2_)_2_]_2_, was purchased from Nanomeps, hexadecylamine (HDA, Aldrich, 98%), oleic acid (OA, 99%, Alfa Aesar), cyclohexane (Aldrich, 99%), igepal CO-520 (Aldrich, 90%), tetraorthosilicate (TEOS, Aldrich, 98%), ammonia solution (28.0–30%, Sigma–Aldrich), poly(ethyleneglycol)monomethyl ether (PEG-OH) (5 kDa, Aldrich), and 3-(triethoxysilyl)propylisocyanate (TESPIC, Alfa Aesar) were used as received. Hexadecylammonium chloride (HDA.HCl) was prepared according to reference [27] from hexadecylamine (HDA, Aldrich, 90%). MilliQ water (18 MΩ) was used for all aqueous preparations. 

### 3.1. Synthesis of Iron NPs (NPFe)

NPFe were prepared according to a published procedure [27]. In a typical experiment 6 mmol of HDA.HCl (1.66 g), 8 mmol of HDA (1.93 g), and 2 mmol of [Fe(N(SiMe_3_)_2_)_2_]_2_ (1.56 g) were dissolved in 80 mL of mesitylene. The reacting medium was heated at 150 °C for 63 h. The particles were separated with an NdFeB magnet (N42, 1.3 T) overnight, and the solvent was pipetted out. The particles were then washed three times with 80mL of toluene (<5 ppm of H_2_O), and three times with 60 mL of dry ethanol (<30 ppm of H_2_O). The obtained powder contained 95%w of iron (as deduced from ICP analysis). Isolated yield: 79% (179 mg of black powder).

### 3.2. Oleic Acid Treatment of the NPFe

In a Fisher-Porter tube 2 mL of oleic acid in 80 mL of toluene were added on 179 mg of NPs, and the dark solution was sonicated for 15 minutes. After one night of magnetic separation with an NdFeB magnet (N42, 1.3 T), the black powder obtained was washed three times with 15 mL of toluene. The material obtained contains 81%w of iron (as deduced from ICP analysis). Isolated yield: 73% (150 mg of powder).

### 3.3. Coating of the Iron NPs with Silica (NPFe@FeOx@SiO_2_)

The procedure was adapted from [30]. Silica coating was obtained by ammonia catalyzed hydrolysis of tetraethoxysilane (TEOS) in the presence of Igepal CO-520. In a 30 mL Pyrex tube, 15 mL of cyclohexane and 0.8 mL of igepal CO-520 were mixed and mechanically stirred for 5 min. A 1.5 mL of cyclohexane solution containing 5.15–5.25 mg of iron NPs was added to the previous solution and mechanically stirred for 30 min. Then, 0.13 mL of a NH_4_OH solution (30%) were added and the solution was mechanically stirred for 15 min. Finally, 0.15 mL of TEOS were added and the solution was mechanically stirred for 15 min, then the reaction was left ongoing without agitation for 48 hours. Addition of 0.118 µL of acetic acid neutralized the pH to stop the catalytic process. Then addition of 10 mL of methanol followed by magnetic separation with an NdFeB magnet (N42, 1.3 T) allowed the recovery of a black powder. This powder was washed one more times with 10 mL of methanol (Vortex 1 min, sonication 5 min, centrifugation 30 min, 9000 rpm, 5 °C), two times with 10 mL of ethanol (Vortex 1min, sonication 5 min, centrifugation 30 min, 9000 rpm, 5 °C), and three times with 10 mL mQ water (Vortex 1 min, sonication 5 min, centrifugation 30 min, 9000 rpm, 5 °C). The final product was dispersed in 10 mL of MilliQ water and stored in a fridge (solution 1). ([NPFe@FeOx@SiO_2_] ≅ 3 mg(10%w_Fe_).mL^−1^).

### 3.4. Synthesis of PEG-Si(OCH_2_CH_3_)_3_

The PEG-silane was prepared according to a published procedure [54] from 5 kDa PEG-OH and TESPIC, and was characterized by NMR (1H, 13C and 29Si): 1H NMR CDCl3 δ(ppm): 5.04 (1H, s), 4,22 (2H, t), 3.84 (6H, q), 3.66 (340 H, s), 3.48 (2H, m), 3.40 (3H, s), 3.18 (2H, m), 1.24 (9H, t), 0.64 (2 H, t). 13C CP MAS solid state NMR, probe 4G, Vr = 6 kHz, δ(ppm): 70.7, 58.3, 43.7, 23.8, 18.4, 7.9. 29Si MAS NMR, rotor of 4 mm, Vr = 6 kHz, δ(ppm): −46.6, and by DRIFT ν(CH2-CH2)= 2872 cm^−1^, ν(C=O) = 1718 cm^−1^, ν(Si-O) = 1110 cm^−1^. DHYD = 4.9 nm (PDI = 11%).

### 3.5. PEGylation of NPFe@FeOx@SiO_2_ (NPFe@FeOx@SiO_2_-PEG)

The procedure was adapted from [54]. Of absolute ethanol 5 mL was added to solution 1 to yield a 2:1 ratio *v/v* solution (MilliQ water:ethanol). Addition of 25 mg of previously synthesized PEG-silane was followed by a 10 minutes sonication of the mixture. Then, 0.325 mL of 30% NH_4_OH was added to the mixture, which was then heated at 50 °C for 48 hours without agitation. The particles were then washed 4 times with 10 mL of MilliQ water (Vortex 1 min, sonication 5 minutes, centrifugation 1 hour 30 minutes, 12000 rpm, 15 °C). The NPs were redispersed in 10 mL of MilliQ water (solution 2). ([NPFe@FeOx@SiO_2_-PEG] ≅ 1.5 mg (11%w_Fe_).mL^−1^). The solution was then lyophilized in order to be analyzed by solid state NMR: ^13^C MAS NMR rotor of 3.2 mm Vr = 10 kHz δ(ppm): 127.6, 70.1, 47.0, 43.0, 28.5, 25.7, 21.0, and by DRIFT: ν(CH_2_–CH_2_) = 2872 cm^−1^, ν(C=O) = 1724 cm^−1^, ν(Si–O–Si) = 1104 cm^−1^. A surface coverage ≅1 PEG/32 nm² was deduced from elemental analysis. The magnetization curve saturated at 110 A.m^-2^.kg_Fe_^−1^ at 310 K.

### 3.6. Synthesis of Silica NPs (NPSiO_2_) and PEGylated-NPSiO_2_ (NPSiO_2_-PEG)

Reported in SI.

### 3.7. Characterizations

Iron content in the samples was determined by inductively coupled plasma-optical emission spectrometry (ICP-OES, PerkinElmer Optima 2100 DV ICP), after digesting the samples into a mixture of HNO_3_: HCl (1:3 ratio *v/v*) and diluting them with ultrapure water.

C, H, and N contents were determined on a ICAP 7600ICP-OES analyzer (ThermoScientific) on a PERKIN ELMER 2400 série II analyzer (ThermoScientific).

IR spectra were recorded in ATR mode on a Bruker Alpha FT-IR spectrophotometer placed in the glove box for air sensitive samples and on a PerkinElmer Frontier FT-IR spectrophotometer for air stable samples. Diffuse reflectance **infrared** Fourier transform (DRIFT) spectra were acquired on a Nexus Nicolet with a DRIFT accessory from Perkin Elmer with a resolution of 0.8 cm^−1^ and a deuterated triglycine sulfate (DTGS) detector. All spectra were recorded in solid state after lyophilization of the samples.

Magic angle spinning (MAS) solid state NMR spectra were recorded on a Bruker Avance IIIHD 400 wide-bore instrument equipped with a 3.2 or 4 mm MAS probe, with the sample rotation frequency being set respectively at 6 kHz or 10 kHz. All ^1^H, ^29^Si, and ^13^C chemical shifts are reported using the δ scale and are referenced to tetramethylsilane (TMS) at 0 ppm. High-power ^1^H decoupling was used to record ^13^C and ^29^Si spectra. All spectra were recorded on lyophilized samples. NPFe@FeOx@SiO_2_ and NPFe@FeOx@SiO_2_-PEG samples were diluted 50 times with silica powder.

Magnetic measurements were performed on a SQuID magnetometer (MPMS Quantum Design). The samples were prepared in gelatine capsules. The saturation magnetization curves were obtained at 310 K in a magnetic field varying from 0 to 50 kOe. 

Transmission electron microscopy (TEM) samples were prepared by the drop casting method on 3.05 mm copper grids of 400 mesh from Pelanne instruments coated with a collodion film thickness of around 20–50 nm. TEM images were recorded on a MET Jeol JEM 1011 and 1400 instrument, size distributions were acquired by measuring a minimum of 250 objects using the open source ImageJ software. Sizes are given as mean ± standard deviation according to a Gaussian fit of the corresponding size distribution. Energy dispersive X-ray (EDX) study has been performed on a MET Jeol JEM 2100F instrument.

The hydrodynamic size was measured by dynamic light scattering (DLS), using a Zetasizer NanoZ device (Malvern instruments). Dilute suspensions were prepared in MilliQ water (10^−5^ mol/L Fe). The solutions were filtrated on a cellulose 0.450 µm filter before the DLS analysis. DHYD in this work refers to the Z-average diameter. 

The Zeta potential study was performed, using a Zetasizer NanoZ device (Malvern instruments). Two stock solutions were prepared for each sample, one for the study in basic pH and one for the study in acidic pH. The pH was adjusted with 0.001 mol.L^−1^ and 0.01 mol.L^−1^ HCl and NaOH solutions in order to keep the dilution of the NPs the same for each measurement. Samples were analyzed from pH = 1 to pH = 12.

To evaluate the efficiency of the hydrophilic suspensions as MRI contrast agents, T_2_ and T_1_ relaxation times were measured with a Minispec MQ60 (Bruker) operating at 37 °C and 60 MHz, with a magnetic field of 1.5T and a Minispec MQ20 (Bruker) operating at 37 °C and 20 MHz with a magnetic field of 0.47 T. The relaxation rate Ri values (1/Ti, s^−1^, I = 1,2), obtained from the relaxation times measured (T_i_, s), were corrected by subtracting the water relaxation rate (R(H_2_O) = 0.2826 s^−1^) in the absence of the contrast agent. Linear fitting of the data related to the iron concentration (mmol.L^−1^) gives straight lines whose slopes are the relaxivities (ri, s^−1^.mmol^−1^, with R² > 0.99). Nuclear magnetic relaxation diffusion (NMRD) profiles were recorded at 37 °C on a field cycling relaxometer (Stelar, Italy) at magnetic fields ranging from 4.7 10^−4^ (0.02 MHz) to 0.94 T (40 MHz). The fitting of the data was performed according to the model developed by Roch et al. [55,56] using a home-made fitting program. It has to be noticed that this model was developed for iron oxide nanoparticles and may thus not be completely appropriate for iron-iron oxide (Fe@FeOx) core-shell architectures. Phantom samples were prepared in 250 µL eppendorfs by diluting the samples (solutions 1 and 2) with pure water to afford the expected concentration range, images were acquired on a biospec 9.4T MRI from Bruker and on an ICON 1T from Bruker.

Toxicity assessments: Human colorectal carcinoma cells (HCT 116) and normal human colon fibroblasts (CCD112-CoN) were obtained from American Type Culture Collection (ATCC, Manassas, VA, USA). Human transformed skin fibroblasts 1BR3G were obtained from European Collection of Authenticated Cell Cultures (ECACC, Public Health England, UK). HCT116 and 1BR3G cells were routinely cultured in Dulbecco’s modified eagle medium (DMEM, Invitrogen) containing 10% heat-inactivated fetal bovine serum and 1% antibiotic-antimycotic solution (Gibco) at 37 °C in a humidified 10% CO_2_ atmosphere. CCD112-CoN cells were maintained in minimum essential medium (MEM, Gibco) containing 10% heat-inactivated fetal bovine serum, 1 mM sodium pyruvate (Gibco) and 1% antibiotic-antimycotic solution. The cytotoxicity activity of each sample was evaluated using the PrestoBlue Cell Reagent (Life Technologies) assay. Stock solutions for NPs were prepared in MilliQ water. All working concentrations were prepared in cell culture media with a maximum of 10% of MilliQ water in all working concentrations including control cells. Cells were plated in 96-well plates at a density of 5 × 10^3^ cells/well in 100 μL of culture medium and were allowed to grown overnight. After this time, cells were treated with different concentrations of each samples during 24 h and 72 h and then 10 μL of PrestoBlue reagent was added following the standard protocol [57]. After 3 h incubation, fluorescence was measured exciting at 531 nm (emission at 572 nm) using a Victor3 multiwell microplate reader (Perkin Elmer). The relative cell viability (%) for each sample related to the control cells without treatment was calculated. Each sample was tested in triplicate.

Hemocompatibility assays: a hemolysis assay of human red blood cells (HRBCs) was performed to test the hemocompatibility of our samples. NPFe@FeOx@SiO_2_ and NPFe@FeOx@SiO_2_-PEG were suspended in Ca^2+^/Mg^2+^-free Dulbecco’s phosphate buffered saline (DPBS, Life Technologies) at a concentration of 200 µg/mL. Heparinized human whole blood obtained from human donors was diluted in DPBS to a hemoglobin concentration of 160 mg/mL. Nanoparticles suspensions and blood preparations were mixed in a 1:1 proportion and incubated at 37 °C for 4 hours in a microtube rotator. Blood preparations were mixed 1:1 with PBS and deionized water as a negative (NC) and positive control (PC) respectively. All conditions were performed in triplicate.

Following the incubation, the samples were centrifuged at 800× *g* for 15 minutes at room temperature. Supernatants were transferred into new tubes and centrifuged at 16,000× *g* for 15 minutes at room temperature to remove the remaining nanoparticles; the supernatants were transferred into a 96-well plate and their absorbance was read at 545 nm in a Victor3 multiwell microplate reader (Perkin Elmer). Hemolytic activity was calculated following Equation (1):

(1) $$\%Haemolysis=\frac{Abs_{Sample}-Abs_{NC}}{Abs_{PC}-Abs_{NC}}.$$

## 4. Conclusions

Nanoparticles comprising a crystalline magnetic iron/iron oxide core surrounded by an amorphous silica shell were successfully synthesized. The ease of their functionalization was exemplified by grafting PEG chains at their surface. The physico-chemical characteristics of these new materials were determined by the use of a set of complementary techniques and their interest as contrast agents for MRI was evaluated, as well as their cytotoxicity on human cell lines (both cancerous and normal). These nanomaterials displayed a good stability in water, no cytotoxicity even after 72 h of incubation on the HCT116 and 1BR3G cell lines, and no hemolytic activity against HRBCs suggesting they might not be toxic in in vivo assays despite the long circulating time generally reported for NPs (more than 75% NPs still present in the body after 48h) [58]. These nanomaterials also induced a much better darkening effect than the T_2_ contrast agent presently marketed for use in MRI. This improved contrast in the images was directly related to the larger magnetization of the material in comparison with the one of the reference material. Indeed, the magnetization of these nanomaterials saturates at lower magnetic fields than iron oxide NPs making them more suited for clinical applications. Their magnetization being higher suggests that good images could be recorded with reduced loading in contrast agent during the injection, even if high field MRI was envisaged. Then, this kind of material could help detect tiny features such as early stage metastases, if properly targeted. Presently, given their hydrodynamic size these nanoparticles could be envisaged for imaging the reticuloendothelial system, any disease state characterized by an acute inflammatory response, or gastrointestinal imaging, or for cell tracking [1,2]. 

Finally, the choice of amorphous silica as a coating shell will allow a fine tuning of the properties of the nanomaterial by adapting the thickness of this shell or by adding targeting groups at the surface, both synthetic strategies that have already been developed for magnetite NPs coated with comparable silica shells [30,31] and are readily available. This facile chemistry thus opens a broad range of applications and is currently investigated in our group. Then more complete toxicity evaluations will be needed before any clinical use can be envisaged.

## Figures and Tables

**Table 1:** Z-average hydrodynamic diameters of NPFe@FeOx@SiO_2_ and NPFe@FeOx@SiO_2_-PEG after 2 weeks and 2 months of settling in MilliQ water.

Sample	NPFe@FeOx@SiO_2_		NPFe@FeOx@SiO_2_-PEG	
Aging	2 weeks	2 months	2 weeks	2 months
Z average	99 nm	172 nm	111 nm	202 nm
PdI	±16%	±30%	±20%	±21%

**Table 2:** Theoretical values obtained from the fitting of the NMRD profiles of NPFe@FeOx@SiO_2_ and NPFe@FeOx@SiO_2_-PEG.

	NPFe@FeOx@SiO_2_	NPFe@FeOx@SiO_2_-PEG
Ms (A.m². kg_MM_)	23.20	20.20
Radius (nm)	13.7	13.5
D (cm². s^−1^)*	3.01 e^-5^	3.01 e^−5^
τ_Néel_ (ns)	40.1	24.3

* Fixed value during the fitting.

**Table 3:** Relaxometry analysis at 37 °C of NPFe@FeOx@SiO_2_, NPFe@FeOx@SiO_2_-PEG, and RESOVIST® at 20 MHz and 60 Hz. Values at 400 MHz were deduced from the contrast observed in the phantom images.

Frequency.	Relaxivity (mM^−1^.s^−1^)	NPs* 2 weeks Old	NPs*-PEG 2 weeks Old	NPs* 2 months Old	NPs*-PEG 2 months Old	RESOVIST® Experimental	DOTAREM® Experimental
20 MHz	r_1_	9.5 ± 0.5	9.8 ± 0.5	8.9 ± 0.4	3.6 ± 0.2	25.0 ± 1.3	4.1 ± 0.2
	r_2_	165.5 ± 8.3	186.5 ± 9.3	163.1 ± 8.2	181.7 ± 9.1	161.8 ± 8.1	4.9 ± 0.2
	r_2_/r_1_	17.5	19	18.3	50	6.5	1.2
60 MHz	r_1_	3.1 ± 0.2	2.5 ± 0.1	3.5 ± 0.2	1.30 ± 0.07	11.0 ± 0.6	3.7 ± 0.2
	r_2_	166.0 ± 8.3	188.0 ± 9.4	175.0 ± 8.8	185.1 ± 9.3	157.8 ± 7.9	4.0 ± 0.2
	r_2_/r_1_	55.3	75.2	50	142	14.3	1.08
400 MHz	r_2_	208.0 ± 10.4/237.0 ± 11.9	254.0 ± 12.7	224.0 ±11.2	239.0 ± 12.0	232.0 ± 11.6	4.7 ± 0.2

NPs*: NPFe@FeOx@SiO_2._

## Supplementary Materials

The following are available online at https://www.mdpi.com/1420-3049/24/24/4629/s1, Complementary data on NPFe, NPFe@FeOx@SiO2, and NPFe@FeOx@SiO2-PEG: Figure S1: Size histograms of (a) toluene washed NPFe (b) ethanol washed NPFe and (c) Oleic acid treated NPFe. Figure S2: IR spectra of NPFe washed with toluene in comparison with that of (a) HDA.HCl and (b) HDA. Figure S3: IR spectra of (a) the dried first toluene supernatant and (b) the dried first ethanol supernatant. Figure S4: Size histograms of (a) iron/iron oxide cores and (b) silica shells of NPFe@FeOx@SiO2 drawn from TEM micrographs. Figure S5: size histograms of (a) iron/iron oxides cores and (b) silica shells of NPFe@FeOx@SiO2-PEG drawn from TEM micrographs. Figure S6: EDX analysis on an NPFe@FeOx@SiO2-PEG and corresponding TEM image recorded in HAADF mode. Figure S7: (a) hydrodynamic diameters measured by DLS (intensity weighted) and b) zeta potential analysis of NPFe@FeOx@SiO2. Figure S8: (a) hydrodynamic diameters measured by DLS (intensity weighted) and B zeta potential analysis of NPFe@FeOx@SiO2-PEG. Figure S9: Phantoms MRI images acquired at (a) 9.4 T, T1 sequences (TR = 750 ms, TE = 10 ms) and (b) at 1 T, T1 sequences (TR = 500 ms, TE = 8 ms). Figure S10: Reproducibility of the cytotoxicity assays of (a) NPFe@FeOx@SiO2 (100 µg/mL) and NPFe@FeOx@SiO2-PEG (100 µg/mL) and (b) NPSiO2 (100 µg/mL) and NPSiO2-PEG (100 µg/mL) on HCT116 cells after 24 hours and 72 hours of incubation at 37 °C. Figure S11: Cytotoxicity assays on HCT116 cells after 72 hours of incubation of (a) NPFe@FeOx@SiO2 and (b) NPFe@FeOx@SiO2-PEG, dose effect. Concentration used for viability assays is outlined in grey. Figure S12: Cytotoxicity assays on 1BR3G cells after 72 hours of incubation of (a) NPFe@FeOx@SiO2 and (b) NPFe@FeOx@SiO2-PEG, dose effect. Concentration used for viability assays is outlined in grey. Figure S13: Cytotoxicity assays on CCD112-CoN cells after 72 hours of incubation of a) NPFe@FeOx@SiO2 and b) NPFe@FeOx@SiO2-PEG, dose effect. Concentration used for viability assays is outlined in grey. Complementary data on NPSiO2 and NPSiO2-PEG: Experimental part. Figure S14: (a) 1H (large signal at 4.65 ppm corresponds to H2O), (b) 13C, and (c) 29Si NMR spectra of PEGylated NPSiO2 (blue) and non PEGylated NPSiO2 (red). Figure S15: DRIFT spectra of NPSiO2 and NPSiO2-PEG. Figure S16: TEM pictures of (a) NPSiO2 and (b) NPSiO2-PEG; scale bar 200 nm. Figure S17: Size histograms of (a) NPSiO2 and (b) NPSiO2-PEG.

## References

[1] Laurent S., Henoumont C., Stanicki D., Boutry S., Lipani E., Belaid S., Muller R.N., Vander Elst L. (2017). MRI Contrast Agents: From Molecules to Particles.

[2] Wahsner J., Gale E.M., Rodríguez-Rodríguez A., Caravan P. (2019). Chemistry of MRI Contrast Agents: Current Challenges and New Frontiers. Chem. Rev..

[3] Ni D., Bu W., Ehlerding E.B., Cai W., Shi J. (2017). Engineering of inorganic nanoparticles as magnetic resonance imaging contrast agents. Chem. Soc. Rev..

[4] Shen Z., Wu A., Chen X. (2017). Iron Oxide Nanoparticle Based Contrast Agents for Magnetic Resonance Imaging. Mol. Pharm..

[5] Vallabani N.V.S., Singh S. (2018). Recent advances and future prospects of iron oxide nanoparticles in biomedicine and diagnostics. 3 Biotech.

[6] Suárez-García S., Arias-Ramos N., Frias C., Candiota A.P., Arús C., Lorenzo J., Ruiz-Molina D., Novio F. (2018). Dual *T*_1_/*T*_2_ Nanoscale Coordination Polymers as Novel Contrast Agents for MRI: A Preclinical Study for Brain Tumor. ACS Appl. Mater. Interfaces.

[7] Tse B.W.-C., Cowin G.J., Soekmadji C., Jovanovic L., Vasireddy R.S., Ling M.-T., Khatri A., Liu T., Thierry B., Russell P.J. (2015). PSMA-targeting iron oxide magnetic nanoparticles enhance MRI of preclinical prostate cancer. NanoMed.

[8] Jafari A., Salouti M., Shayesteh S.F., Heidari Z., Rajabi A.B., Boustani K., Nahardani A. (2015). Synthesis and characterization of Bombesin-superparamagnetic iron oxide nanoparticles as a targeted contrast agent for imaging of breast cancer using MRI. Nanotechnology.

[9] Hayashi K., Nakamura M., Sakamoto W., Yogo T., Miki H., Ozaki S., Abe M., Matsumoto T., Ishimura K. (2013). Superparamagnetic Nanoparticle Clusters for Cancer Theranostics Combining Magnetic Resonance Imaging and Hyperthermia Treatment. Theranostics.

[10] Wu M., Zhang D., Zeng Y., Wu L., Liu X., Liu J. (2015). Nanocluster of superparamagnetic iron oxide nanoparticles coated with poly (dopamine) for magnetic field-targeting, highly sensitive MRI and photothermal cancer therapy. Nanotechnology.

[11] Revia R.A., Zhang M. (2016). Magnetite nanoparticles for cancer diagnosis, treatment, and treatment monitoring: Recent advances. Mater. Today.

[12] Glaria A., Soulé S., Hallali N., Ojo W.S., Mirjolet M., Fuks G., Cornejo A., Allouche J., Dupin J.C., Martinez H. (2018). Silica coated iron nanoparticles: Synthesis, interface control, magnetic and hyperthermia properties.

[13] Wang C.M., Baer D.R., Thomas L.E., Amonette J.E., Antony J., Qiang Y., Duscher G. (2005). Void formation during early stages of passivation: Initial oxidation of iron nanoparticles at room temperature. J. Appl. Phys..

[14] Miguel O.B., Gossuin Y., Morales M.P., Gillis P., Muller R.N., Veintemillas-Verdaguer S. (2007). Comparative analysis of the ^1^H NMR relaxation enhancement produced by iron oxide and core-shell iron–iron oxide nanoparticles. Magn. Reson. Imaging.

[15] Cheong S., Ferguson P., Feindel K.W., Hermans I.F., Callaghan P.T., Meyer C., Slocombe A., Su C.-H., Cheng F.-Y., Yeh C.-S. (2011). Simple Synthesis and Functionalization of Iron Nanoparticles for Magnetic Resonance Imaging. Angew. Chem. Int. Ed..

[16] Lacroix L.-M., Frey Huls N., Ho D., Sun X., Cheng K., Sun S. (2011). Stable Single-Crystalline Body Centered Cubic Fe Nanoparticles. Nano Lett..

[17] Hadjipanayis C.G., Bonder M.J., Balakrishnan S., Wang X., Mao H., Hadjipanayis G.C. (2008). Metallic Iron Nanoparticles for MRI Contrast Enhancement and Local Hyperthermia. Small.

[18] Ferguson P.M., Feindel K.W., Slocombe A., MacKay M., Wignall T., Delahunt B., Tilley R.D., Hermans I.F. (2013). Strongly Magnetic Iron Nanoparticles Improve the Diagnosis of Small Tumours in the Reticuloendothelial System by Magnetic Resonance Imaging. PLoS ONE.

[19] Yoon T.-J., Lee H., Shao H., Weissleder R. (2011). Highly Magnetic Core-Shell Nanoparticles with a Unique Magnetization Mechanism. Angew. Chem. Int. Ed..

[20] Zhou Z., Sun Y., Shen J., Wei J., Yu C., Kong B., Liu W., Yang H., Yang S., Wang W. (2014). Iron/iron oxide core/shell nanoparticles for magnetic targeting MRI and near-infrared photothermal therapy. Biomaterials.

[21] Masoudi A., Madaah Hosseini H.R., Seyed Reyhani S.M., Shokrgozar M.A., Oghabian M.A., Ahmadi R. (2012). Long-term investigation on the phase stability, magnetic behavior, toxicity, and MRI characteristics of superparamagnetic Fe/Fe-oxide core/shell nanoparticles. Int. J. Pharm..

[22] Dumestre F., Chaudret B., Amiens C., Renaud P., Fejes P. (2004). Superlattices of Iron Nanocubes Synthesized from Fe[N(SiMe_3_)_2_]_2_. Science.

[23] Margeat O., Respaud M., Amiens C., Lecante P., Chaudret B. (2010). Ultrafine metallic Fe nanoparticles: Synthesis, structure and magnetism. Beilstein J. Nanotechnol..

[24] Lacroix L.-M., Lachaize S., Falqui A., Blon T., Carrey J., Respaud M., Dumestre F., Amiens C., Margeat O., Chaudret B. (2008). Ultrasmall iron nanoparticles: Effect of size reduction on anisotropy and magnetization. J. Appl. Phys..

[25] Lacroix L.-M., Lachaize S., Falqui A., Respaud M., Chaudret B. (2009). Iron Nanoparticle Growth in Organic Superstructures. J. Am. Chem. Soc..

[26] Margeat O., Dumestre F., Amiens C., Chaudret B., Lecante P., Respaud M. (2005). Synthesis of iron nanoparticles: Size effects, shape control and organisation. Prog. Solid State Chem..

[27] Meffre A., Lachaize S., Gatel C., Respaud M., Chaudret B. (2011). Use of long chain amine as a reducing agent for the synthesis of high quality monodisperse iron(0) nanoparticles. J. Mater. Chem..

[28] Caltagirone C., Bettoschi A., Garau A., Montis R. (2015). Silica-based nanoparticles: A versatile tool for the development of efficient imaging agents. Chem. Soc. Rev..

[29] Chen F., Hableel G., Zhao E.R., Jokerst J.V. (2018). Multifunctional nanomedicine with silica: Role of silica in nanoparticles for theranostic, imaging, and drug monitoring. J. Colloid Interface Sci..

[30] Ding H.L., Zhang Y.X., Wang S., Xu J.M., Xu S.C., Li G.H. (2012). Fe_3_O_4_@SiO_2_ Core/Shell Nanoparticles: The Silica Coating Regulations with a Single Core for Different Core Sizes and Shell Thicknesses. Chem. Mater..

[31] Chen F., Bu W., Chen Y., Fan Y., He Q., Zhu M., Liu X., Zhou L., Zhang S., Peng W. (2009). A Sub-50-nm Monosized Superparamagnetic Fe_3_O_4_@SiO_2_ T_2_-Weighted MRI Contrast Agent: Highly Reproducible Synthesis of Uniform Single-Loaded Core-Shell Nanostructures. Chem. Asian J..

[32] Glorani G., Marin R., Canton P., Pinto M., Conti G., Fracasso G., Riello P. (2017). Pegylated silica nanoparticles: Cytotoxicity and macrophage uptake. J. Nanoparticle Res..

[33] Karakoti A.S., Das S., Thevuthasan S., Seal S. (2011). PEGylated Inorganic Nanoparticles. Angew. Chem. Int. Ed..

[34] Herman D.A.J., Ferguson P., Cheong S., Hermans I.F., Ruck B.J., Allan K.M., Prabakar S., Spencer J.L., Lendrum C.D., Tilley Richard D. (2011). Hot-injection synthesis of iron/iron oxide core/shell nanoparticles for T_2_ contrast enhancement in magnetic resonance imaging. Chem. Commun..

[35] Schumaker N.E., Garland C.W. (1970). Infrared Investigation of Structural and Ordering Changes in Ammonium Chloride and Bromide. J. Chem. Phys..

[36] Fredrickson L.R., Decius J.C. (1977). The Raman spectrum of the ordered phase of NH_4_Cl and ND_4_Cl: Dipole and polarizability derivatives. J. Chem. Phys..

[37] Gharbi K. (2017). Elaboration de Nanoparticules d’or et de fer pour des Applications Biomédicales. Ph.D. Thesis.

[B38-molecules-24-04629] 38.Accordingly, the IR spectrum of the dried ethanol supernatant showed the presence of NH_4_Cl (3114 cm^−1^, 3003 cm^−1^, 2816 cm^−1^, 1390 cm^−1^), and aliphatic chains (2916 cm^−1^ and 2849 cm^−1^) attributed to residual HAD·HCl (Figure SI 3b).

[39] Trunova A.V., Meckenstock R., Barsukov I., Hassel C., Margeat O., Spasova M., Lindner J., Farle M. (2008). Magnetic characterization of iron nanocubes. J. Appl. Phys..

[40] Branca M., Marciello M., Ciuculescu-Pradines D., Respaud M., Morales M.d.P., Serra R., Casanove M.-J., Amiens C. (2015). Towards MRI T_2_ contrast agents of increased efficiency. J. Magn. Magn. Mater..

[41] Toney M., Davenport A., Oblonsky L., Ryan M., Vitus C. (1997). Atomic Structure of the Passive Oxide Film Formed on Iron. Phys. Rev. Lett..

[42] Wang C., Baer D.R., Amonette J.E., Engelhard M.H., Antony J., Qiang Y. (2009). Morphology and Electronic Structure of the Oxide Shell on the Surface of Iron Nanoparticles. J. Am. Chem. Soc..

[43] Signorini L., Pasquini L., Savini L., Carboni R., Boscherini F., Bonetti E., Giglia A., Pedio M., Mahne N., Nannarone S. (2003). Size-dependent oxidation in iron/iron oxide core-shell nanoparticles. Phys. Rev. B.

[44] Liz-Marzán L.M., Giersig M., Mulvaney P. (1996). Synthesis of Nanosized Gold−Silica Core−Shell Particles. Langmuir.

[B45-molecules-24-04629] 45.EDX study confirmed the silica shell / iron rich core architecture (Figure S6). We assume that TEOS is not fully condensed at this stage, based on ^29^Si MAS NMR studies carried out on NPSiO_2_ taken as a reference (see supplementary information for details).

[46] Joshi H.M., De M., Richter F., He J., Prasad P.V., Dravid V.P. (2013). Effect of silica shell thickness of Fe_3_O_4_ –SiOx core–shell nanostructures on MRI contrast. J. Nanoparticle Res..

[47] Perry J.L., Reuter K.G., Kai M.P., Herlihy K.P., Jones S.W., Luft J.C., Napier M., Bear J.E., DeSimone J.M. (2012). PEGylated PRINT Nanoparticles: The Impact of PEG Density on Protein Binding, Macrophage Association, Biodistribution, and Pharmacokinetics. Nano Lett..

[48] Lee N., Hyeon T. (2012). Designed synthesis of uniformly sized iron oxide nanoparticles for efficient magnetic resonance imaging contrast agents. Chem. Soc. Rev..

[49] Koenig S.H., Kellar K.E. (1995). Theory of 1/T_1_ and 1/T_2_ NMRD profiles of solutions of magnetic nanoparticles. Magn. Reson. Med..

[50] Pinho S.L.C., Laurent S., Rocha J., Roch A., Delville M.-H., Mornet S., Carlos L.D., Vander Elst L., Muller R.N., Geraldes C.F.G.C. (2012). Relaxometric Studies of γ-Fe_2_O_3_@SiO_2_ Core Shell Nanoparticles: When the Coating Matters. J. Phys. Chem. C.

[51] Khurshid H., Hadjipanayis C.G., Chen H., Li W., Mao H., Machaidze R., Tzitzios V., Hadjipanayis G.C. (2013). Core/shell structured iron/iron-oxide nanoparticles as excellent MRI contrast enhancement agents. J. Magn. Magn. Mater..

[52] Harisinghani M.G., Barentsz J., Hahn P.F., Deserno W.M., Tabatabaei S., van de Kaa C.H., de la Rosette J., Weissleder R. (2003). Noninvasive Detection of Clinically Occult Lymph-Node Metastases in Prostate Cancer. N. Engl. J. Med..

[53] McGrath A.J., Dolan C., Cheong S., Herman D.A.J., Naysmith B., Zong F., Galvosas P., Farrand K.J., Hermans I.F., Brimble M. (2017). Stability of polyelectrolyte-coated iron nanoparticles for T_2_-weighted magnetic resonance imaging. J. Magn. Magn. Mater..

[54] He Q., Zhang J., Shi J., Zhu Z., Zhang L., Bu W., Guo L., Chen Y. (2010). The effect of PEGylation of mesoporous silica nanoparticles on nonspecific binding of serum proteins and cellular responses. Biomaterials.

[55] Roch A., Muller R.N., Gillis P. (1999). Theory of proton relaxation induced by superparamagnetic particles. J. Chem. Phys..

[56] Laurent S., Forge D., Port M., Roch A., Robic C., Vander Elst L., Muller R.N. (2008). Magnetic Iron Oxide Nanoparticles: Synthesis, Stabilization, Vectorization, Physicochemical Characterizations, and Biological Applications. Chem. Rev..

[57] Xu M., McCanna D.J., Sivak J.G. (2015). Use of the viability reagent PrestoBlue in comparison with alamarBlue and MTT to assess the viability of human corneal epithelial cells. J. Pharmacol. Toxicol. Methods.

[58] Li Y.-F., Chen C. (2011). Fate and Toxicity of Metallic and Metal-Containing Nanoparticles for Biomedical Applications. Small.

