# Supplementary material for: Silica Coated Iron/Iron Oxide Nanoparticles as a Nano-Platform for T2 Weighted Magnetic Resonance Imaging
Source: Molecules. 2019 Dec 17;24(24):4629. doi: 10.3390/molecules24244629 (PMC6943426; doi:10.3390/molecules24244629)
Supplement: Supplementary file 1 [file molecules-24-04629-s001.pdf]

Supporting information

Silica coated iron/iron oxide nanoparticles as a nano-platform for T_2 weighted magnetic resonance imaging.

Paul Mathieu[1, 2], Yannick Coppel[1,2], Marc Respaud[3], Quyen T. Nguyen[1,2], Sébastien Boutry[4,5], Sophie Laurent[4,5], Dimitri Stanicki[4], Céline Henoumont[4], Fernando Novio[6], Julia Lorenzo [7], David Montpeyó [7], Catherine Amiens[1, 2*]

[1] CNRS, LCC (Laboratoire de Chimie de Coordination), 205 route de Narbonne, BP 44099, F-31077 Toulouse Cedex 4, France.
[2] Université de Toulouse, UPS, INPT, F-31077 Toulouse Cedex 4, France.
[3] LPCNO, INSA, 135 Avenue de Rangueil, 31077 Toulouse Cedex 4, France.
[4] Department of General, Organic and Biomedical Chemistry, NMR and Molecular Imaging Laboratory, University of Mons, 19 avenue Maistriau, B- 7000 Mons, Belgium.
[5] Center for Microscopy and Molecular Imaging (CMMI), Université de Mons (UMONS), Charleroi, Belgium.
[6] Departament de Química, Universitat Autònoma de Barcelona (UAB), Campus UAB, 08193 Cerdanyola del Vallès, Barcelona, Spain.
[7] Institut de Biotecnologia i Biomedicina, Departament de Bioquimica i de Biologia Molecular, Universitat Autonoma de Barcelona, 08193 Bellaterra, Spain.

* Correspondence: catherine.amiens@lcc-toulouse.fr; Tel.: +33 561333182 (C.A.)

Complementary data on NPFe, NPFe@FeOx@SiO2 and NPFe@FeOx@SiO2-PEG systems.

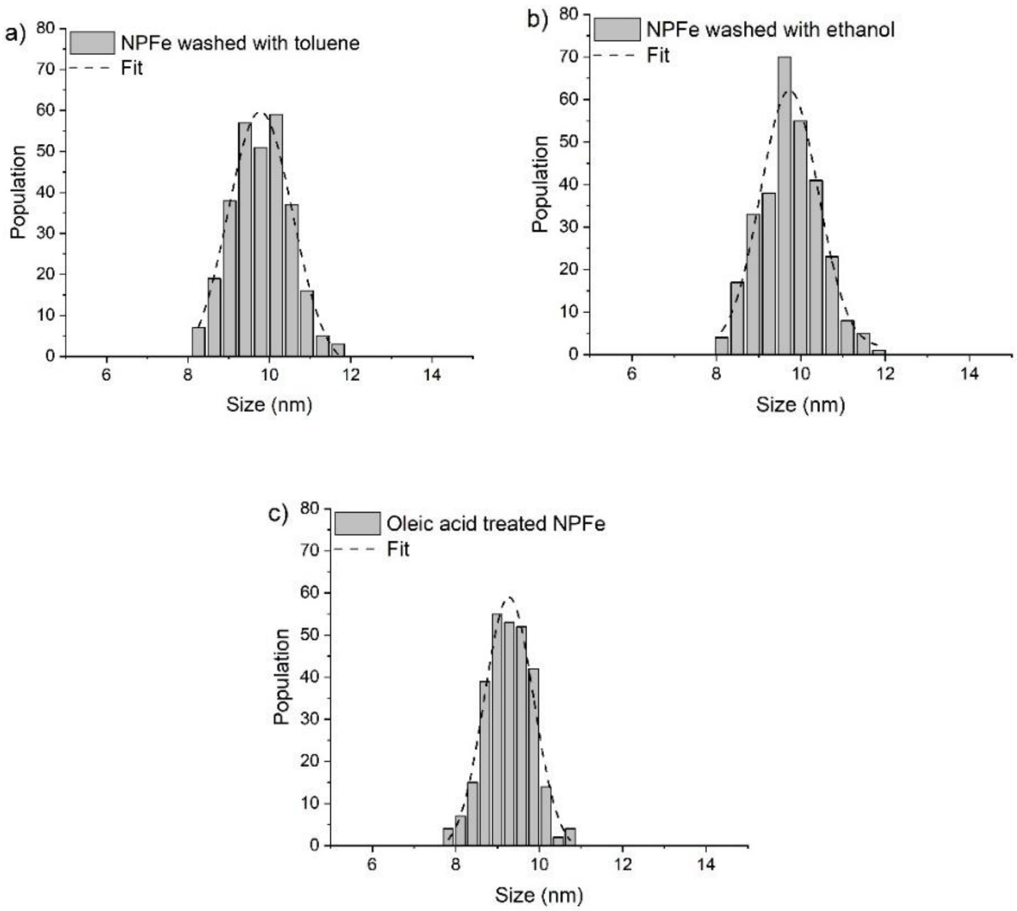

Figure S1. Size histograms of a) toluene washed NPFe b) ethanol washed NPFe and c) oleic acid treated NPFe.

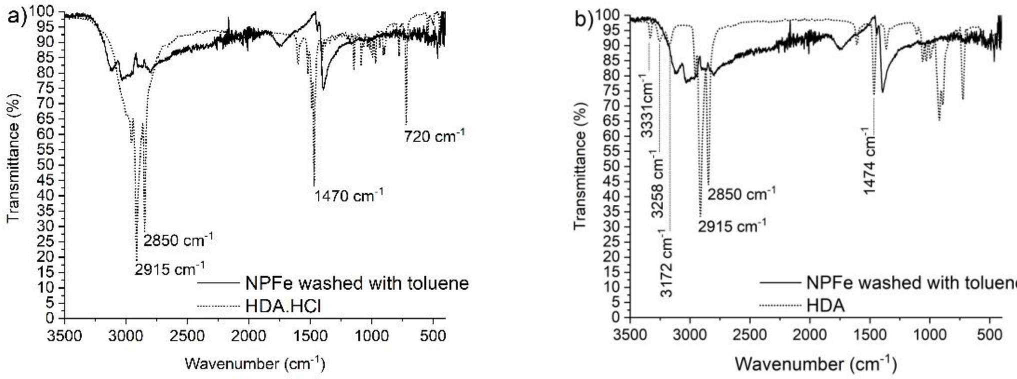

Figure S2. IR spectra of NPFe washed with toluene in comparison with that of a) HDA.HCl and b) HDA.

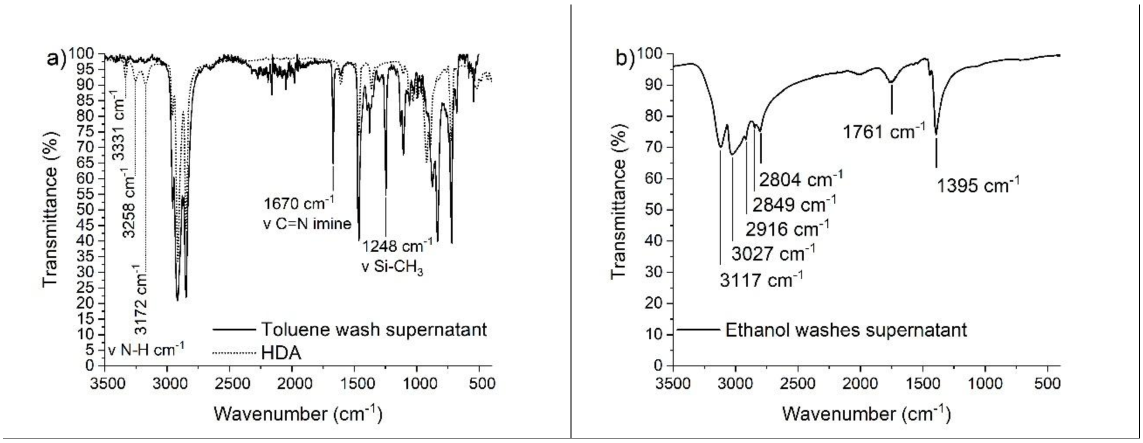

Figure S3. IR spectra of a) the dried first toluene supernatant and b) the dried first ethanol supernatant.

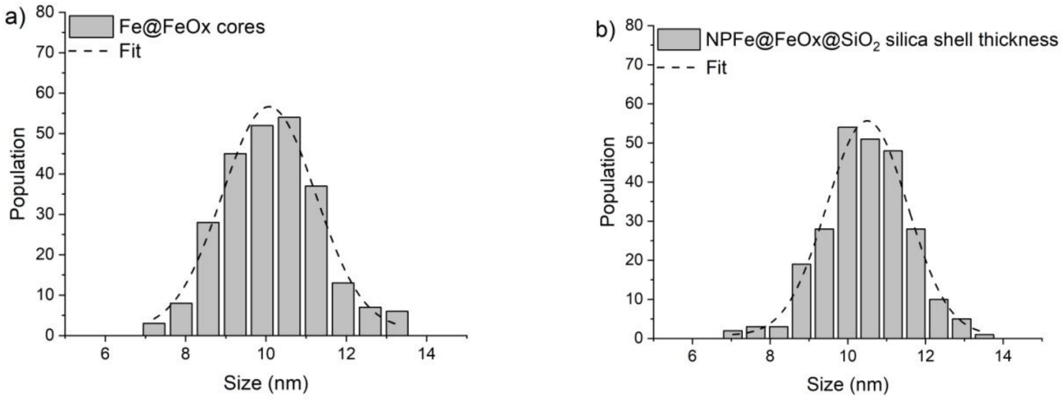

Figure S4. Size histograms of a) Fe@FeOx cores and b) silica shells of NPFe@FeOx@SiO_2 drawn from TEM micrographs.

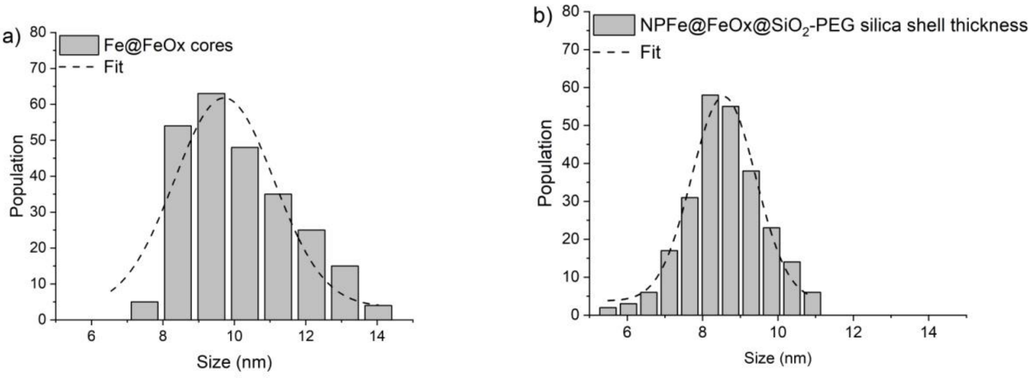

Figure S5. Size histograms of a) Fe@FeOx cores and b) silica shells of NPFe@FeOx@SiO_2-PEG drawn from TEM micrographs.

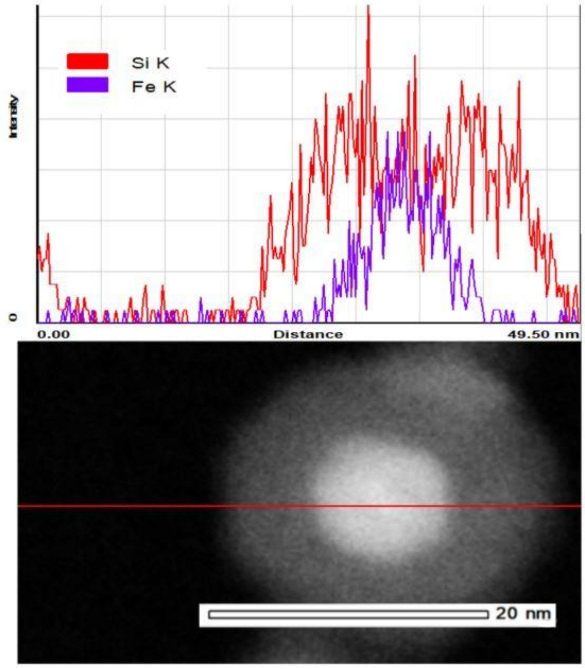

Figure S6. EDX analysis on a NP fromNPFe@FeOx@SiO_2-PEG sample and corresponding TEM image recorded in HAADF mode.

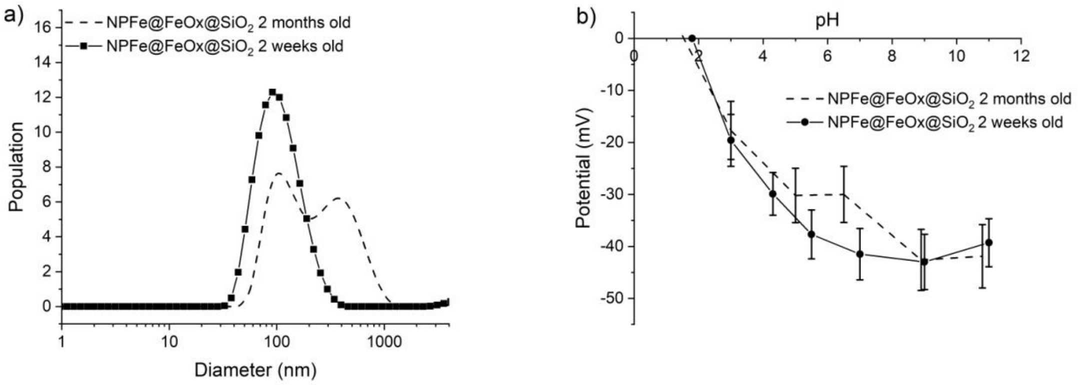

Figure S7. a) Hydrodynamic diameters measured by DLS (intensity weighted) and b) zeta potential analysis of NPFe@FeOx@SiO_2.

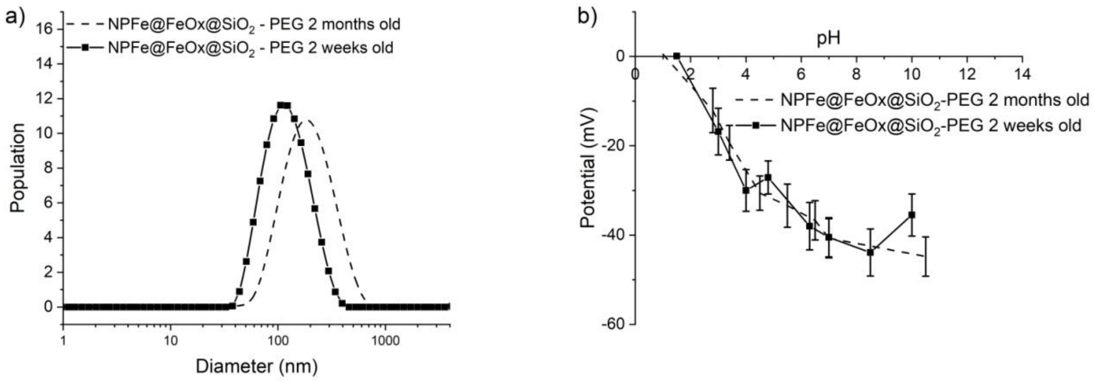

Figure S8. a) Hydrodynamic diameters measured by DLS (intensity weighted) and b) zeta potential analysis of NPFe@FeOx@SiO_2-PEG.

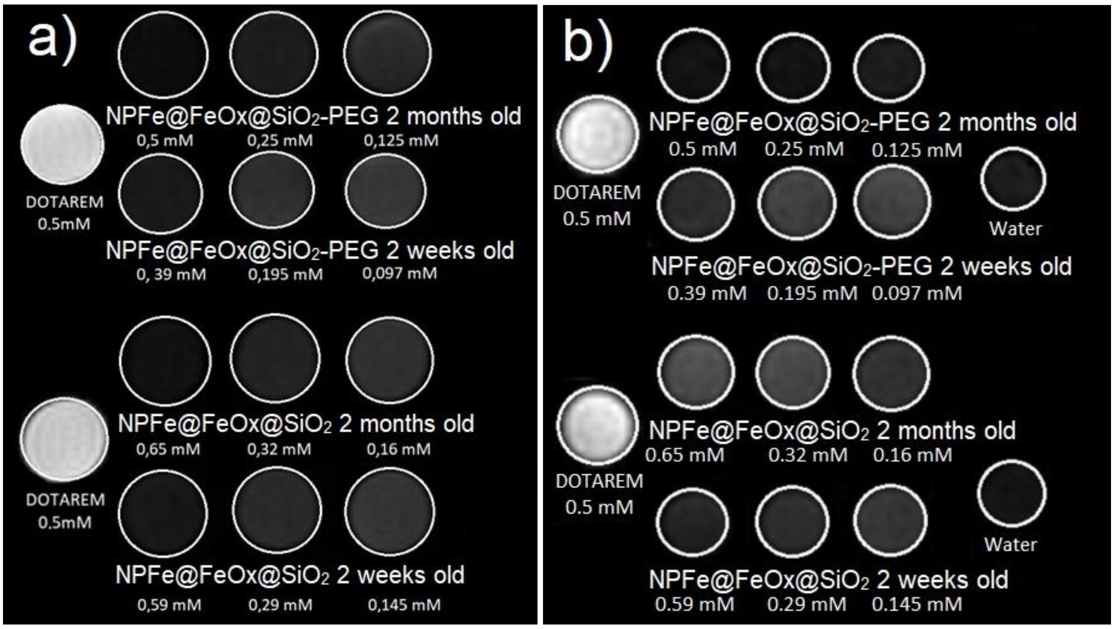

Figure S9. Phantoms MRI images acquired at a) 9.4T, T_1 sequences (TR :750 ms, TE = 10ms) and b) at 1T, T_1 sequences (TR = 500 ms, TE = 8 ms).

Cytotoxicity assessments :

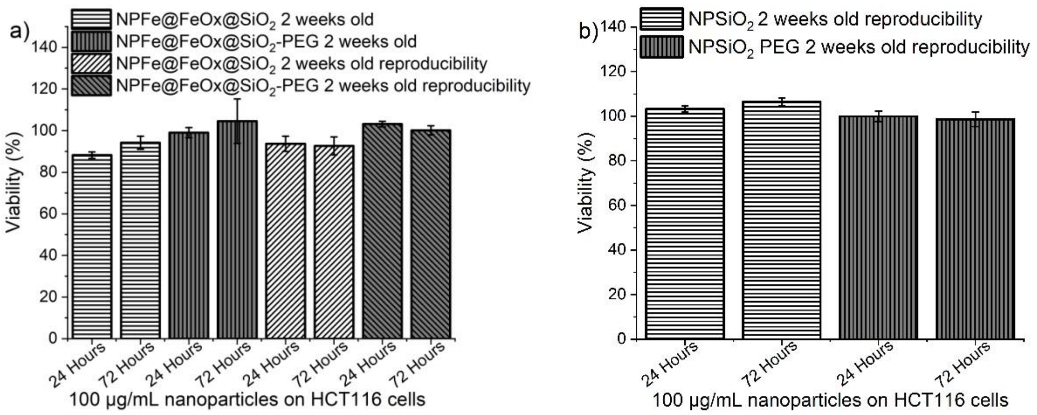

Figure S10. Reproducibility of the cytotoxicity assays of a) NPFe@FeOx@SiO_2 (100µg/mL) and NPFe@FeOx@SiO_2-PEG (100µg/mL) and b) NPSiO_2 (100µG/mL) and NPSiO_2-PEG (100µG/mL) on HCT116 cells after 24 hours and 72 hours of incubation at 37°C.

Dose effect on viability was studied after 72h of incubation, as it is generally observed that NPs are not excreted and stay for a long period in the body (more than 75% still present after 48h and some still detectable after months) [58].

In these conditions, HCT116 and 1BR3G cells resisted high NPs loading whatever the core and surface state of the NPs (Figures 11 and 12). If PEGylation slightly enhanced the viability of the 1BR3G cells, this effect was more significant with the more sensitive CCD112-CoN cells (Figure 14). Note that the potentially less toxic 2 months aged samples were not tested at high concentration with this cell line.

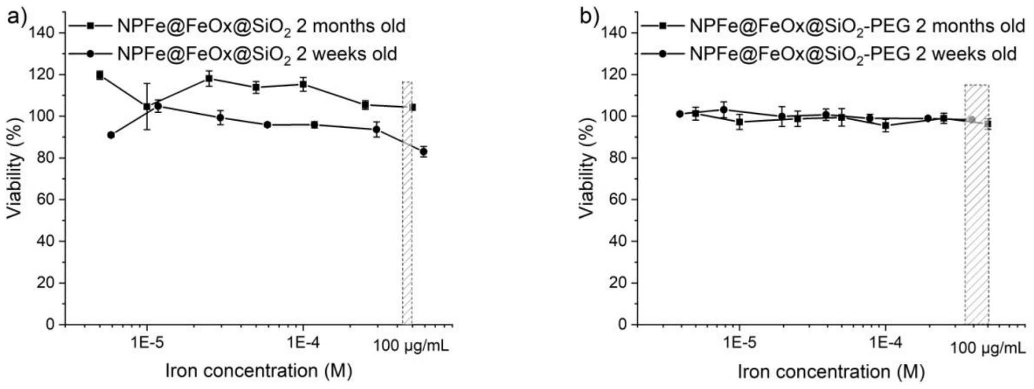

Figure S11. Cytotoxicity assays on HCT 116 cells after 72 hours of incubation of a) NPFe@FeOx@SiO_2 and b) NPFe@FeOx@SiO_2-PEG, dose effect. Concentration used for the viability assays is outlined in grey.

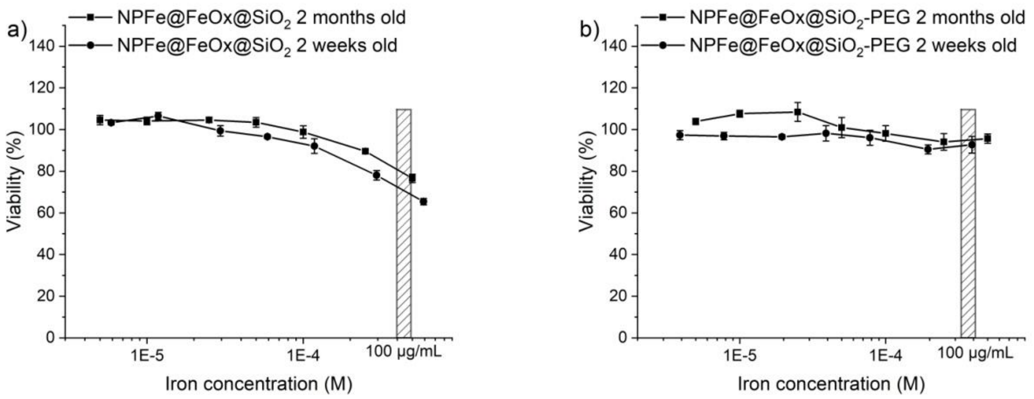

Figure S12. Cytotoxicity assays on 1BR3G cells after 72 hours of incubation of a) NPFe@FeOx@SiO_2 and b) NPFe@FeOx@SiO_2-PEG, dose effect. Concentration used for the viability assays is outlined in grey.

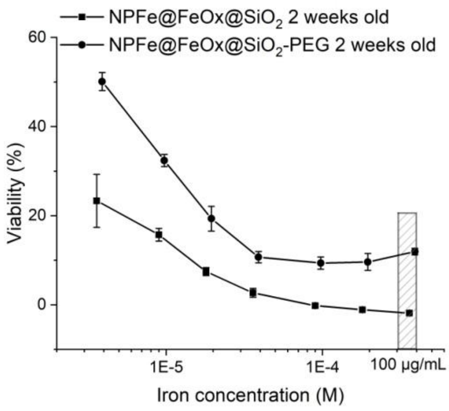

Figure S13. Cytotoxicity assays on CCD112-CoN cells after 72 hours of incubation of NPFe@FeOx@SiO_2 (squares) and NPFe@FeOx@SiO_2-PEG (dots), dose effect. Concentration used for the viability assays is outlined in grey.

Table S1. Haemolytic activity of NPFe@FeOx@SiO_2 and NPFe@FeOx@SiO_2-PEG samples.

Condition	Haemolytic activity (%)
**50% dH_2O**	100% ± 1.18%
**DPBS**	0% ± 0,03%
**NPFe@FeOx@SiO_2**	0.46% ± 0,10%
**NPFe@FeOx@SiO_2-PEG**	0.07% ± 0,04%

Synthesis and characterization of $NPSiO_2$ and $NPSiO_2$-PEG samples

Experimental part :

Methanol (96% STREM), ethanol (99.9% Aldrich), cyclohexane (Aldrich, 99%), igepal CO-520 (Aldrich, 90%), tetraorthosilicate (TEOS, Aldrich, 98%), ammonia solution (28.0-30%, SigmaAldrich), acetic acid (98% Aldrich), poly(ethyleneglycol)monomethyl ether (PEG-OH) (5kDa, Aldrich) were used as received. MilliQ water (18 MΩ) was used for all aqueous preparations.

Synthesis of silica NPs ($NPSiO_2$), adapted from [30] : Silica NPs were prepared by ammonia catalyzed hydrolysis of tetraethoxysilane (TEOS) in the presence of Igepal CO-520. 16.5mL of cyclohexane and 0.8mL of Igepal CO-520 were placed in a glasstube and mechanically stirred for 30min. Then, 0.13mL of a NH_4OH solution (30%) was added and the solution was mechanically stirred for 15min. Finally, 0.15mL of TEOS was added and the solution was mechanically stirred for 15 minutes and the reaction was left ongoing without agitation for 48 hours. Addition of 0.118µL of acetic acid neutralizes the pH to stop the catalytic process. 10 mL of methanol (Vortex 1min, sonication 5min, centrifugation 30min, 9000 rpm, 5°C), three times with 10 mL of ethanol (Vortex 1min, sonication 5min, centrifugation 30min, 9000 rpm, 5°C), and three times with 10 mL milliQ water (Vortex 1min, sonication 5min, centrifugation 30min, 9000 rpm, 5°C). The final product was dispersed in 10 mL of milliQ water and stored in a fridge (solution 1-SiO2). ([$NPSiO_2$]≅ 2.5mg.mL^{-1}). The powder was then lyophilized and analyzed by solid state ^{13}C NMR (empty) and by DRIFT: ν(Si-O-Si) = 1104 cm^{-1}.

Synthesis of PEGylated-$NPSiO_2$ ($NPSiO_2$-PEG) adapted from [54]: After the synthesis of the silica NPs, 5mL of absolute ethanol was added to solution 1-SiO_2 to create 15 mL of a 2:1 v/v ratio (milliQ water:ethanol) solution. Addition of 25 mg of previously synthesized PEG-silane was followed by a 10 minutes sonication of the mixture. Then, 0.325 mL of 30% NH_4OH were then added to the mixture and then heated at 50°C for 48 hours without agitation. The particles were then washed 4 times with 10 mL of milliQ water (Vortex 1 min, sonication 5 minutes, centrifugation 1 hour 30 minutes, 12000 rpm, 15°C). The NPs were redispersed in 10 mL of milliQ water (solution 2-SiO_2). ([$NPSiO_2$-PEG] ≅ 1 mg.mL^{-1} and ≅1PEG /28nm^2). The powder was then lyophilized in order to be analyzed by solid state NMR and DRIFT. ^{13}C MAS NMR probe 3.2mm, Vr= 8 KHz, δ(ppm) = 79.59.

The silica NPs were prepared following the protocol established for the coating of the NPFe except for the absence of the magnetic NPs. Their average size was determined by TEM (Figure SI 16-17) and DLS (23,02 +/- 3,6 nm and 158 +/- 15 nm respectively), and their degree of condensation was investigated from their ^{29}Si CPMAS NMR spectrum. Q2, Q3 and Q4 signals corresponding respectively to Si nuclei in $(RO)_2Si(OSi)_2$, $(RO)Si(OSi)_3$ and $Si(OSi)_4$ environments could be observed. ^{29}Si MAS NMR spectrum evidenced a major contribution from Q3 ones (Q2 (9%), Q3 (50%), and Q4 (41%)) which clearly showed that TEOS wasn't fully condensed at the end of the reaction. Absence of signals in the regions for aromatic hydrogen and carbon atoms evidenced the complete removal of igepal and the purity of the product. The IR spectrum of the $NPSiO_2$ was recorded in DRIFT mode to increase the signal over noise ratio (Figure SI 15). It displayed mainly a large peak around 1093 cm^{-1} attributed to Si-O vibration bands, and an absorption at 1650cm^{-1} indicative, with the large hump above 3000 cm^{-1}, of the presence of adsorded water. The weak signal at circa 2960 cm^{-1} could be attributed to ν(C-H) vibrations of the ethoxy groups in agreement with the NMR study. PEGylation of the NPs was achieved by reacting 5kDa PEG chains, previously modified to accomodate a triethoxysilane function, with $NPSiO_2$ in a 1/1 v/v ethanol/water mixture for 48h at 50°C. Condensation of the triethoxysilane end group onto the silica surface was catalyzed by ammonia. After extensive purification, the white product obtained was analyzed by NMR, IR spectroscopy, TEM and DLS, and the data were compared to those recorded from $NPSiO_2$. The ^{29}Si CPMAS NMR spectra (Figure SI 14c,d) showed T type signals in the -70 to -80 ppm area indicating the presence of Si-C

bonds in the powder of the PEGylated NPs, while none was observed from pure NP SiO_2. The amount of PEG grafting is rather weak and only a slight difference in the ratios of the Q2, Q3, and Q4 contributions determined from ^{29}Si MAS NMR before (Q2 2%, Q3 47%, Q4 51%) and after (Q2 3%, Q3 42%, Q4 55%) the PEG grafting, could be observed. The peak at 3.75 ppm in ^{1}H MAS (Figure SI 14a) and at 69.6 ppm in the ^{13}C MAS (Figure SI 14b) NMR spectra could be attributed to the O-C<u>H</u>$_2$ group of the PEG chains grafted at the surface of the NPs. The ^{13}C signal at 29.3 ppm and ^{1}H signal in the aliphatic region (1.3 ppm to 0 ppm) could correspond to the C and H of the propyl chain (possibly also to grease). ^{1}H signals above 4.6 ppm account for SiOH groups and physisorbed water molecules. These NMR analysis brought a first proof of the PEG grafting on pure NP SiO_2.

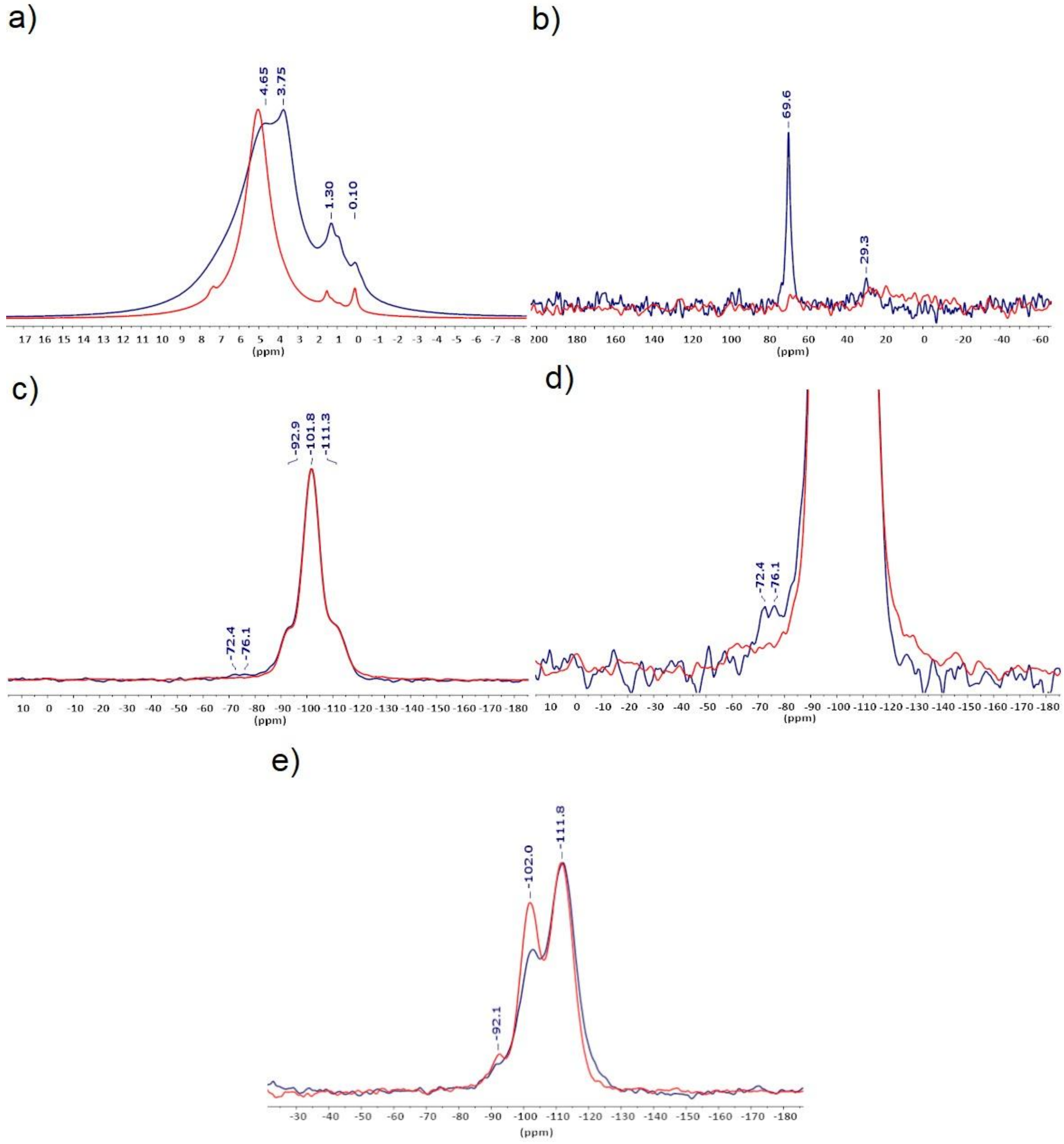

Figure S14. a) ^{1}H(large signal at 4.65ppm corresponds to <u>H</u>$_2$O), b) ^{13}C, c) and d) ^{29}Si NMR spectra of PEGylated NPSiO$_2$ (blue) and non PEGylated NPSiO$_2$ (red), e) ^{29}Si MAS NMR spectra of PEGylated NPSiO$_2$ (blue) and non PEGylated NPSiO$_2$ (red)

In comparison with the spectrum recorded from $NPSiO_2$, DRIFT analysis of the PEGylated NPs showed new C-H stretches at 2872 cm^{-1} characteristic of the PEG chains. Both NMR and IR analysis thus pointed to a successful grafting of the PEG at the surface of the silica NPs.

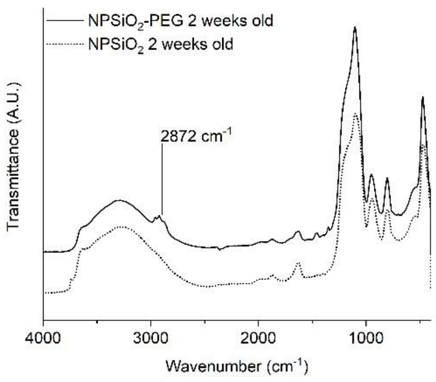

Figure S15. : DRIFT spectra of $NPSiO_2$ and $NPSiO_2$ – PEG.

Elemental analysis of the NPs before and after PEGylation evidenced a 2%w of PEG in the final powder. This percentage corresponds to a grafting density of 1 PEG/28 nm^2. TEM analysis was finally carried out to investigate any change in the morphology of the NPs during the grafting process. TEM images (Figure SI 16) didn't evidence any significant change in the average diameter (23.8 +/- 2.7 nm after PEGylation). DLS showed an average size of 118.7 +/- 10.1 nm.

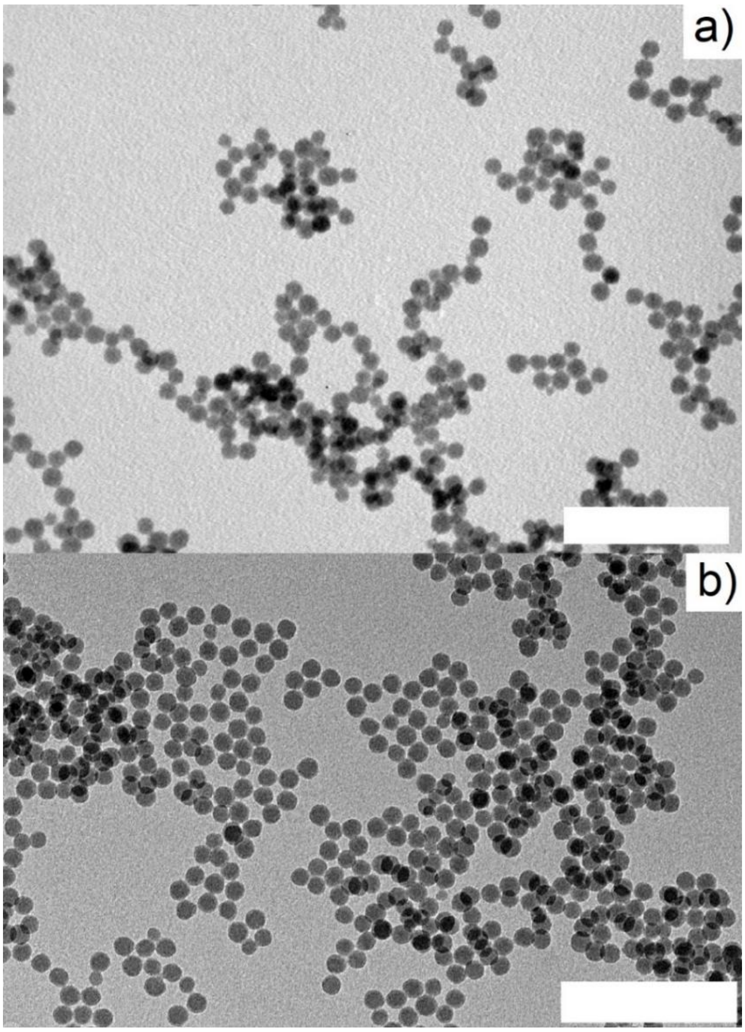

Figure S16. TEM pictures of a) $NPSiO_2$ and b) $NPSiO_2$-PEG; scale bar 200nm.

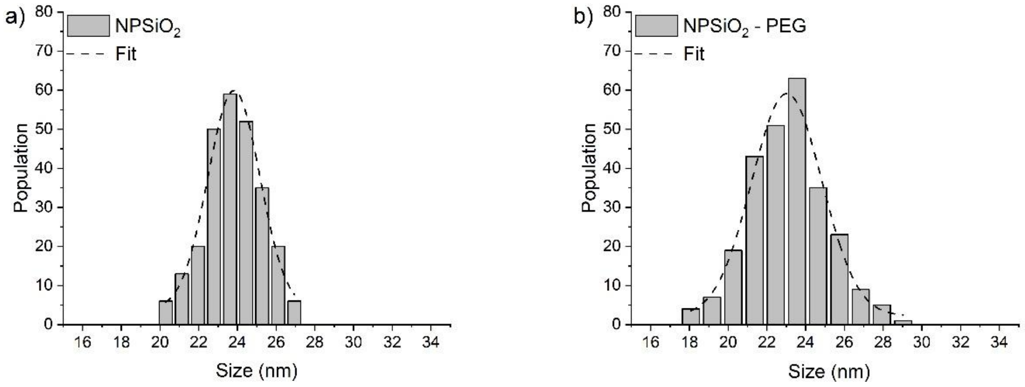

Figure S17. Size histograms of a) $NPSiO_2$ and b) $NPSiO_2$-PEG.
